# A Decision-Making Model with Cloud Model, Z-Numbers, and Interval-Valued Linguistic Neutrosophic Sets

**DOI:** 10.3390/e26110892

**Published:** 2024-10-22

**Authors:** Huakun Chen, Jingping Shi, Yongxi Lyu, Qianlei Jia

**Affiliations:** 1 School of Automation, Northwestern Polytechnical University, Xi’an 710072, China; shijingping@nwpu.edu.cn (J.S.); yongxilyu@nwpu.edu.cn (Y.L.); jiaql@mail.nwpu.edu.cn (Q.J.); 2 Shaanxi Province Key Laboratory of Flight Control and Simulation Technology, Xi’an 710072, China

**Keywords:** interval-valued linguistic neutrosophic sets (IVLNSs), Z-numbers, trapezium cloud model, Z-interval-valued linguistic neutrosophic set-trapezium–trapezium cloud (Z-IVLNS-TTC), group decision-making

## Abstract

Interval-valued linguistic neutrosophic sets (IVLNSs), Z-numbers, and the trapezium cloud model are powerful tools for expressing uncertainty and randomness. This paper aims to combine these methodologies. First, we review relevant concepts and operators, introducing a novel combination of IVLNSs and Z-numbers, which establishes a new form of expression. Subsequently, we propose the Z-interval-valued linguistic neutrosophic set-trapezium–trapezium cloud (Z-IVLNS-TTC) model, designed to minimize information loss and distortion during quantification. A novel method for calculating the objective weight vector is then developed using multi-objective programming (MOP). Drawing inspiration from the TOPSIS method, we propose a new approach for calculating the distance between Z-IVLNS-TTCs based on the p-norm. Finally, a group decision-making problem is presented to demonstrate the practical application of the proposed method. To validate the effectiveness and feasibility of the method, sensitivity analysis and comparisons with existing approaches are conducted.

## 1. Introduction

In 1965, fuzzy sets (FSs) were introduced by Zadeh [1] to express uncertainty and randomness. Subsequently, various theories emerged, including Atanassov’s intuitionistic fuzzy sets (AIFSs) [2,3], Atanassov’s interval-valued intuitionistic fuzzy sets (AIVIFSs) [4,5], triangle intuitionistic fuzzy sets (TIFSs) [6], and Atanassov’s interval-valued intuitionistic linguistic numbers (AIVILNs) [7,8]. However, these approaches still face challenges in real-world scenarios. For instance, when an expert evaluates a new antique, they might estimate its authenticity as 0.6, its fakeness as 0.2, and the uncertainty as 0.1. To address such issues, neutrosophic sets were proposed [9], offering a more robust framework with three parameters: membership degree $T_{A}\left(x\right)$, uncertainty degree $I_{A}\left(x\right)$, and non-membership degree $F_{A}\left(x\right)$.

In recent years, neutrosophic sets have been extensively developed. To enhance the capabilities, interval-valued neutrosophic sets (IVNSs) were introduced, allowing parameters to be represented as intervals [10]. This was followed by the definition of single-valued neutrosophic sets (SVNSs) and simplified neutrosophic sets (SNSs), along with relevant mathematical operations. These concepts were then applied to the multicriteria group decision-making (MCGDM) problems [11]. Also, multi-valued neutrosophic sets, expected value, and Hamming distance were introduced in [12]. Researchers have also explored the nature, operations, and measurement. To aggregate different neutrosophic sets, simplified aggregation operators like the simplified neutrosophic weighted averaging (SNWA) operator and the simplified neutrosophic weighted geometric (SNWG) operator were proposed in [13]. The single-valued neutrosophic number-weighted averaging (SVNNWA) operator and single-valued neutrosophic number-weighted geometric (SVNNWG) operator were developed based on the Archimedean t-conorm and t-norm in [14]. Furthermore, the simplified neutrosophic number power ordered weighted averaging (SNNPOWA) operator and simplified neutrosophic number power ordered weighted geometric (SNNPOWG) operator were introduced in [15]. To describe neutrosophic sets, the score function, accuracy function, and distance degree were defined in [16,17].

In addition to the neutrosophic sets, Z-numbers [18] proposed by Zadeh are another significant concept that deserves attention. A typical Z-number is in the form of Z=(A,B), where *A* and *B* represent fuzzy restriction and reliability, respectively [19,20]. Given the advantages of Z-numbers in representing uncertain information, scholars have focused on this field [21,22,23]. Aliev [24] introduced generalized decision theories into Z-numbers. Later, an approximate reasoning method was proposed based on the rule “IF-THEN” and Z-numbers [25]. To develop Z-numbers, a novel concept called Z-Advanced number was introduced [26]. Yaakob [27] proposed a fuzzy network by combining Z-numbers and TOPSIS method. An extended fuzzy logic was put forward and applied to deal with the problem of information reliability in logical reasoning [28]. Z-numbers were combined with cloud models in [29,30]. Furthermore, Atanassov’s interval-valued intuitionistic fuzzy sets (AIVIFSs) and Z-numbers were integrated in [31].

After sorting out a large amount of the literature, it can be found that there are some deficiencies in the traditional methods. The key contributions of this study can be summarized. Compared with the other fuzzy sets, the interval-valued linguistic neutrosophic sets (IVLNSs) and Z-numbers are characterized by a stronger ability to reveal real-life information. As such, this study synthesizes the two theories and develops a new expression $Z=\left(\left(\left[x_{A}^{L},x_{A}^{U}\right],\left[T_{A}^{L},T_{A}^{U}\right],\left[I_{A}^{L},I_{A}^{U}\right],\left[F_{A}^{L},F_{A}^{U}\right]\right),\left(\left[x_{B}^{L},x_{B}^{U}\right],\left[T_{B}^{L},T_{B}^{U}\right],\left[I_{B}^{L},I_{B}^{U}\right],\left[F_{B}^{L},F_{B}^{U}\right]\right)\right)$, which is more in line with the actual situation. The newly proposed method, while reliable and convincing, is complex and computationally demanding, limiting its practical application. To address this, the trapezium cloud model is employed to simplify calculations and facilitate the conversion between qualitative concepts and quantitative values. Additionally, a new expression, Z-interval-valued linguistic neutrosophic set-trapezium–trapezium cloud (Z-IVLNS-TTC), is introduced. A crucial part of the proposed method is to determine the objective weight vector. After analyzing the previous studies, a novel weight calculation approach based on multi-objective programming (MOP) is proposed. For MCGDM problems, the key step is to calculate the distances between alternatives and ideal solutions using the TOPSIS method. This paper introduces a new method to calculate the distance between Z-IVLNS-TTCs based on the p-norm. It is worth mentioning that this paper is an improved research based on the reference [31], mainly for the expression of information and the calculation method of weights. Additionally, the distance measure and aggregation operator are also improved. In the comparison section, we also compare the algorithm in this paper with the method in [31].

This paper’s structure is as follows: In Section 2, some related concepts are reviewed. In Section 3, the concept of Z-IVLNS-TTC is defined. In Section 4, the power aggregation operators are developed, meanwhile, the methods of calculating weight vector and distance are obtained. In Section 5, the proposed method is applied to a MCGDM problem. In Section 6, the comparison analysis is conducted. The conclusion is given in Section 7.

## 2. Preliminaries

### 2.1. Linguistic Term Sets

**Definition 1**  ([21])**.** *Let $H=\{h_{j}|j=0,1,2,\cdots ,2t,|t\in N\}$ be linguistic term sets. $h_{j}$ represents a possible value for a linguistic variable. $h_{i}$ and $h_{j}$ should satisfy.*

(1) $h_{i}\leq h_{j}$ if and only if i≤j;
(2) The negation operation $neg\left(h_{i}\right)=h_{j}$ if i+j=2t;
(3) If i≥j, then $\max\left\{h_{i},h_{j}\right\}=h_{i}$;
(4) If i≥j, then $\min\left\{h_{i},h_{j}\right\}=h_{j}$.

**Definition 2**  ([32])**.** *For the linguistic term $h_{j}$ in $H=\{h_{j}|j=0,\;1,\;\cdots ,\;2t,\;t\in N\}$, if $\theta_{j}\in \left[0,\;1\right]$ is a numerical value, then the function $f:h_{j}\to \theta_{j}$, which conducts the mapping from $h_{j}$ to $\theta_{j}$.*

(1) $$\theta_{j}=\left\{\begin{array}{ccc} \frac{a^{t}-a^{t-j}}{2a^{t}-2}, & \; & 0\leq j\leq t\\ \frac{a^{t}+a^{j-t}-2}{2a^{t}-2}, & \; & t<j\leq 2t \end{array}\right.$$

### 2.2. Cloud Model

**Definition 3**  ([31,33])**.** *Assume U is the universe of discourse and T denotes the qualitative concept in U. If x∈U is a random instantiation of concept T, which satisfies $x∼N(Ex,En^{\prime 2})$, $En^{\prime 2}∼N\left(En,He^{2}\right)$, and the certainty degree f(x) belonging to T is a membership, which satisfies,*

(2) $$\begin{array}{c} f\left(x\right)=e^{\frac{{\left(x-Ex\right)}^{2}}{2{\left(En^{\prime }\right)}^{2}}}. \end{array}$$

The distribution of *x* in *U* is called a normal cloud and (x,y) is regarded as the cloud droplet. In general, Ex indicates the expected value of cloud droplets, and the parameter acts as the core content of linguistic information. Moreover, the uncertainty and randomness of qualitative concepts are expressed by the entropy En and the hyper entropy He.

**Definition 4**  ([31])**.** *The trapezium cloud model, an extension of normal cloud model, is provided with a stronger ability to solve linguistic information due to the use of interval-valued expected value $\left[\underline{Ex},\bar{Ex}\right]$. All cloud droplets satisfy the following formula.*

(3) $$f\left(x\right)=\left\{\begin{array}{c} e^{-{\left(x-\underline{Ex}\right)}^{2}/\left(2En^{\prime 2}\right)},\;x<\underline{Ex}\\ \;\;1,\;\;\;\;\;\underline{Ex}\leq x\leq \bar{Ex}\\ e^{-{\left(x-\bar{Ex}\right)}^{2}/\left(2En^{\prime 2}\right)},\;x>\bar{Ex} \end{array}\right.$$

Obviously, the trapezium cloud model will be simplified to a normal cloud model when $\underline{Ex}=\bar{Ex}$.

### 2.3. Neutrosophic Sets

**Definition 5.**  *For single-valued linguistic neutrosophic set $A=\{\langle x,T_{A}\left(x\right),I_{A}\left(x\right),F_{A}\left(x\right)\rangle |x\in X\}$, the hesitancy degree of x to A is $\pi_{A}=1+I_{A}\left(x\right)-T_{A}\left(x\right)-F_{A}\left(x\right)$. $T_{A}\left(x\right)$, $I_{A}\left(x\right)$, and $F_{A}\left(x\right)$ represent the membership degree, uncertainty degree, and non-membership degree of x to A.*

**Definition 6.**  *Different from the single-valued linguistic neutrosophic set, the interval-valued linguistic neutrosophic set (IVLNS) is the improvement of the former. $T_{A}\left(x\right)$, $I_{A}\left(x\right)$, and $F_{A}\left(x\right)$ are offered in the form of interval, i.e., $A=\{\langle x,\left[T_{A}^{L}\left(x\right),T_{A}^{U}\left(x\right)\right],\left[I_{A}^{L}\left(x\right),I_{A}^{U}\left(x\right)\right],\left[F_{A}^{L}\left(x\right),F_{A}^{U}\left(x\right)\right]\rangle |x\in X\}$.*

**Definition 7.**  *Given any two IVLNS A and B, if the following conditions are satisfied, then A is called to be included in or equal to B, recorded as A⊆B.*

$$infT_{A}\left(x\right)\geq infT_{B}\left(x\right),\;supT_{A}\left(x\right)\leq supT_{B}\left(x\right)\;infI_{A}\left(x\right)\leq infI_{B}\left(x\right),\;supI_{A}\left(x\right)\geq supI_{B}\left(x\right)\;infF_{A}\left(x\right)\leq infF_{B}\left(x\right),\;supF_{A}\left(x\right)\geq supF_{B}\left(x\right)$$

**Definition 8.**  *For $A=\{\langle x,\left[T_{A}^{L}\left(x\right),T_{A}^{U}\left(x\right)\right],\left[I_{A}^{L}\left(x\right),I_{A}^{U}\left(x\right)\right],\left[F_{A}^{L}\left(x\right),F_{A}^{U}\left(x\right)\right]\rangle |x\in X\}$, the interval-valued hesitancy degree of x to A is $\pi_{A}=\left[1+I^{L}-T^{U}-F^{U},1+I^{U}-T^{L}-F^{L}\right]$.*

### 2.4. Z-Numbers

**Definition 9.**  *A Z-number is employed to describe the association with the real-valued uncertain variable X, recorded as Z=(X,A,B). A denotes the uncertainty constraint on the variable X and B is the reliability measurement of A.*

## 3. Uncertain Z-Numbers and Relevant Concepts

### 3.1. Uncertain Z-Numbers

**Definition 10.**  *Let X be a real-valued uncertain variable, a Z-number with IVLNSs can be shown as $Z^{*}=\left(\left(\left[A^{L},A^{U}\right],\left[T_{A}^{L},T_{A}^{U}\right],\left[I_{A}^{L},I_{A}^{U}\right]\left[F_{A}^{L},F_{A}^{U}\right]\right),\left(\left[B^{L},B^{U}\right],\left[T_{B}^{L},T_{B}^{U}\right],\left[I_{B}^{L},I_{B}^{U}\right],\left[F_{B}^{L},F_{B}^{U}\right]\right)\right)$, where $A^{L}$, $A^{U}$, $B^{L}$, and $B^{U}$ are represented by linguistic terms.*

### 3.2. Conversion Method from Uncertain Z-Numbers to Trapezium Clouds

Although the newly proposed concept is a favorable solution for uncertain problems, an inevitable obstacle is how to quantify the form of evaluation information. Considering that the cloud model makes the transition between qualitative concepts and quantitative values achievable, the trapezium cloud model is adopted in this paper. Suppose that the linguistic term set is $H=\{h_{0},h_{1},h_{2},h_{3},h_{4},h_{5},h_{6},h_{7},h_{8}\}$, a novel approach to transform IVLNSs $A=\{\langle x,\left[h_{i},h_{j}\right],\left[T^{L},T^{U}\right],\left[I^{L},I^{U}\right],\left[F^{L},F^{U}\right]\rangle |x\in X\}$ into trapezium cloud $T=(\underline{He},\underline{En},\left[\underline{Ex},\bar{Ex}\right],\bar{En},\bar{He})$ is introduced [30,31].

(1) Calculate $\theta_{i}$, $\theta_{j}$, $Ex_{i}$, and $Ex_{j}$.

Let the effective domain be $U=[X_{\min},X_{\max}]$, then calculate $\theta_{i}$ and $\theta_{j}$ by (1). $Ex_{i}$ and $Ex_{j}$ can be calculated by $Ex_{i}=X_{\min}+\theta_{i}\left(X_{\max}-X_{\min}\right)$ and $Ex_{j}=X_{\min}+\theta_{j}\left(X_{\max}-X_{\min}\right)$.

(2) Calculate $\underline{Ex}$ and $\bar{Ex}$.

For $A=\{\langle x,\left[h_{i},h_{j}\right],\left[T^{L},T^{U}\right],\left[I^{L},I^{U}\right],\left[F^{L},F^{U}\right]\rangle |x\in X\}$, the interval-valued hesitancy degree is $\pi_{A}=\left[1+I^{L}-T^{U}-F^{U},1+I^{U}-T^{L}-F^{L}\right]$. The average hesitancy degree that reflects the uncertainty of the expected value in the trapezium cloud is $1+\frac{I^{L}+I^{U}-\left(T^{L}+T^{U}+F^{L}+F^{U}\right)}{2}$. Hence,

(4) $$\begin{array}{c} \begin{array}{cc} {\underline{Ex}}_{i} & =Ex_{i}-\left[\frac{1}{2}+\frac{I^{L}+I^{U}-\left(T^{L}+T^{U}+F^{L}+F^{U}\right)}{4}\right]Ex_{i}=\left[\frac{1}{2}-\frac{I^{L}+I^{U}-\left(T^{L}+T^{U}+F^{L}+F^{U}\right)}{4}\right]Ex_{i};\\ {\bar{Ex}}_{i} & =Ex_{i}+\left[\frac{1}{2}+\frac{I^{L}+I^{U}-\left(T^{L}+T^{U}+F^{L}+F^{U}\right)}{4}\right]Ex_{i}=\left[\frac{3}{2}+\frac{I^{L}+I^{U}-\left(T^{L}+T^{U}+F^{L}+F^{U}\right)}{4}\right]Ex_{i}; \end{array} \end{array}$$

(5) $$\begin{array}{c} \begin{array}{cc} \underline{Ex}=\frac{{\underline{Ex}}_{i}+{\bar{Ex}}_{i}}{2} & =\left[1-\frac{I^{L}+I^{U}-\left(T^{L}+T^{U}+F^{L}+F^{U}\right)}{4}\right]Ex_{i}; \end{array} \end{array}$$

Similarly,

(6) $$\begin{array}{c} \begin{array}{c} \bar{Ex}=\left[1-\frac{I^{L}+I^{U}-\left(T^{L}+T^{U}+F^{L}+F^{U}\right)}{4}\right]Ex_{j}. \end{array} \end{array}$$

(3) Calculate $En_{j}$.

From (3), it appears that the distribution of $\left\{x|x\leq \underline{Ex}\right\}$ is a left normal cloud and the distribution of $\left\{x|x\geq \underline{Ex}\right\}$ is a right normal cloud; therefore, $x∼N\left({\underline{Ex}}_{j},\;En_{j}^{\prime 2}\right)$, $En_{j}^{\prime }∼N\left(En_{j},\;He_{j}^{2}\right)$ for $x\leq {\underline{Ex}}_{j}$ and $x∼N\left({\bar{Ex}}_{j},\;En_{j}^{\prime 2}\right)$, $En_{j}^{\prime }∼N\left(En_{j},\;He_{j}^{2}\right)$ for $x\geq {\bar{Ex}}_{j}.$ According to the 3δ principle of the normal distribution, we should comply with $3\;En_{j}^{\prime }=\max\left\{X_{\max}-{\bar{Ex}}_{j},\;{\underline{Ex}}_{j}-X_{\min}\right\}$.

(7) $$\begin{array}{c} \underline{En}=\frac{Ex_{i}-Ex_{i-1}}{3};\\ \bar{En}=\frac{Ex_{j+1}-Ex_{j}}{3}. \end{array}$$

(4) Calculate $He_{j}$.

The He satisfy the 3δ principle of the normal distribution. Therefore,

(8) $$\begin{array}{c} \begin{array}{c} \underline{He}=\frac{\max\{\max\left\{\underline{En_{i}^{\prime }}\right\}-\underline{En_{i}},\underline{En_{i}}-\min\left\{\underline{En_{i}^{\prime }}\right\}\}}{3};\\ \bar{He}=\frac{\max\{\max\left\{\bar{En_{j}^{\prime }}\right\}-\bar{En_{j}},\bar{En_{j}}-\min\left\{\bar{En_{j}^{\prime }}\right\}\}}{3}. \end{array} \end{array}$$

**Definition 11.**  *Given $H=\{h_{0},h_{1},\cdots ,h_{2k},k\in N\}$ and $S=\{s_{0},s_{1},\cdots ,h_{2k^{\prime }},k^{\prime }\in N\}$. If the evaluation information is $\left(\left(\left[h_{i},h_{j}\right],\left[T_{h}^{L},T_{h}^{U}\right]\left[I_{h}^{L},I_{h}^{U}\right],\left[F_{h}^{L},F_{h}^{U}\right]\right),\left(\left[s_{i},s_{j}\right],\left[T_{s}^{L},T_{s}^{U}\right]\left[I_{s}^{L},I_{s}^{U}\right]\left[F_{s}^{L},F_{s}^{U}\right]\right)\right)$, then $Y=(\left(\underline{He},\underline{En},\left[\underline{Ex},\bar{Ex}\right],\bar{En},\bar{He}\right),\left(\underline{he},\underline{en},\left[\underline{ex},\bar{ex}\right],\bar{en},\bar{he}\right))$ can be acquired, which is defined as the Z-interval-valued linguistic neutrosophic set-trapezium–trapezium cloud (Z-IVLNS-TTC).*

## 4. Fundamentals of Z-IVLNS-TTCs

### 4.1. Arithmetic Operators of Z-IVLNS-TTCs

**Definition 12.**  *Let $Z_{i}=\left(\left(\underline{He_{i}},\underline{En_{i}},\left[\underline{Ex_{i}},\bar{Ex_{i}}\right],\bar{En_{i}},\bar{He_{i}}\right),\left(\underline{he_{i}},\underline{en_{i}},\left[\underline{ex_{i}},\bar{ex_{i}}\right],\bar{en_{i}},\bar{he_{i}}\right)\right)$ and $Z_{j}=\left(\left(\underline{He_{j}},\underline{En_{j}},\left[\underline{Ex_{j}},\bar{Ex_{j}}\right],\bar{En_{j}},\bar{He_{j}}\right),\left(\underline{he_{j}},\underline{en_{j}},\left[\underline{ex_{j}},\bar{ex_{j}}\right],\bar{en_{j}},\bar{he_{j}}\right)\right)$, the arithmetic operators of A and B can be summarized as follows.*

(1) $\lambda Z_{i}=\left(\left(\sqrt{\lambda }\underline{He_{i}},\sqrt{\lambda }\underline{En_{i}},\left[\lambda \underline{Ex_{i}},\lambda \bar{Ex_{i}}\right],\sqrt{\lambda }\bar{En_{i}},\sqrt{\lambda }\bar{He_{i}}\right),\left(\underline{he_{i}},\underline{en_{i}},\left[\underline{ex_{i}},\bar{ex_{i}}\right],\bar{en_{i}},\bar{he_{i}}\right)\right)$;

(2) $Z_{k}=Z_{i}⊕Z_{j}=\left(\left(\sqrt{{\underline{He_{i}}}^{2}+{\underline{He_{j}}}^{2}},\sqrt{{\underline{En_{i}}}^{2}+{\underline{En_{j}}}^{2}},\left[\underline{Ex_{i}}+\underline{Ex_{j}},\bar{Ex_{i}}\;+\;\bar{Ex_{j}}\right],\;\;\sqrt{{\bar{En_{i}}}^{2}\;\;\;+\;{\bar{En_{j}}}^{2}},\;\;\sqrt{{\bar{He_{i}}}^{2}\;\;\;+\;{\bar{He_{j}}}^{2}}\right),\;\;\left(\sqrt{\frac{Ex_{i}{\underline{he_{i}}}^{2}+Ex_{j}{\underline{he_{j}}}^{2}}{Ex_{i}+Ex_{j}}},\sqrt{\frac{Ex_{i}{\underline{en_{i}}}^{2}+Ex_{j}{\underline{en_{j}}}^{2}}{Ex_{i}+Ex_{j}}},\left[\frac{Ex_{i}\underline{ex_{i}}+Ex_{j}\underline{ex_{j}}}{Ex_{i}+Ex_{j}},\frac{Ex_{i}\bar{ex_{i}}+Ex_{j}\bar{ex_{j}}}{Ex_{i}+Ex_{j}}\right],\sqrt{\frac{Ex_{i}{\bar{en_{i}}}^{2}+Ex_{j}{\bar{en_{j}}}^{2}}{Ex_{i}+Ex_{j}}},\sqrt{\frac{Ex_{i}{\bar{he_{i}}}^{2}+Ex_{j}{\bar{he_{j}}}^{2}}{Ex_{i}+Ex_{j}}}\right)\right)$;
  where $Ex_{i}=\frac{1}{2}\left(\underline{Ex_{i}}+\bar{Ex_{i}}\right)$ and $Ex_{j}=\frac{1}{2}\left(\underline{Ex_{j}}+\bar{Ex_{j}}\right)$.

(3)
$Z_{k}=Z_{i}⊗Z_{j}=\left(\left(\sqrt{{\left(\underline{He_{i}}\underline{Ex_{j}}\right)}^{2}+{\left(\underline{He_{j}}\underline{Ex_{i}}\right)}^{2}},\sqrt{{\left(\underline{En_{i}}\underline{Ex_{j}}\right)}^{2}+{\left(\underline{En_{j}}\underline{Ex_{i}}\right)}^{2}},\left[\underline{Ex_{i}}\underline{Ex_{j}},\bar{Ex_{i}}\bar{Ex_{j}}\right],\sqrt{{\left(\bar{En_{i}}\bar{Ex_{j}}\right)}^{2}+{\left(\bar{En_{j}}\bar{Ex_{i}}\right)}^{2}},\sqrt{{\left(\bar{He_{i}}\bar{Ex_{j}}\right)}^{2}+{\left(\bar{He_{j}}\bar{Ex_{i}}\right)}^{2}}\right),\left(\sqrt{{\left(\underline{he_{i}}\underline{ex_{j}}\right)}^{2}+{\left(\underline{he_{j}}\underline{ex_{i}}\right)}^{2}},\sqrt{{\left(\underline{en_{i}}\underline{ex_{j}}\right)}^{2}+{\left(\underline{en_{j}}\underline{ex_{i}}\right)}^{2}},\left[\underline{ex_{i}}\underline{ex_{j}},\bar{ex_{i}}\bar{ex_{j}}\right],\sqrt{{\left(\bar{en_{i}}\bar{ex_{j}}\right)}^{2}+{\left(\bar{en_{j}}\bar{ex_{i}}\right)}^{2}},\sqrt{{\left(\bar{he_{i}}\bar{ex_{j}}\right)}^{2}+{\left(\bar{he_{j}}\bar{ex_{i}}\right)}^{2}}\right)\right);$

(4) $Z_{i}^{\lambda }=\left(\left(\sqrt{\lambda }{\underline{Ex_{i}}}^{\lambda -1}\underline{He_{i}},\sqrt{\lambda }{\underline{Ex_{i}}}^{\lambda -1}\underline{En_{i}},\left[{\underline{Ex_{i}}}^{\lambda },{\bar{Ex_{i}}}^{\lambda }\right],\sqrt{\lambda }{\bar{Ex_{i}}}^{\lambda -1}\bar{En_{i}},\sqrt{\lambda }{\bar{Ex_{i}}}^{\lambda -1}\bar{He_{i}}\right),\left(\sqrt{\lambda }{\underline{ex_{i}}}^{\lambda -1}\underline{he_{i}},\sqrt{\lambda }{\underline{ex_{i}}}^{\lambda -1}\underline{en_{i}},\left[{\underline{ex_{i}}}^{\lambda },{\bar{ex_{i}}}^{\lambda }\right],\sqrt{\lambda }{\bar{ex_{i}}}^{\lambda -1}\bar{en_{i}},\sqrt{\lambda }{\bar{ex_{i}}}^{\lambda -1}\bar{he_{i}}\right)\right)$.

**Property 1.**  *Given three Z-IVLNS-TTCs $Z_{i}$, $Z_{j}$, $Z_{k}$, and λ, $\lambda_{1}$, $\lambda_{2}>0$.*

(1) $Z_{i}⊕Z_{j}=Z_{j}⊕Z_{i}$;
(2) $\left(Z_{i}⊕Z_{j}\right)⊕Z_{k}=Z_{i}⊕\left(Z_{j}⊕Z_{k}\right)$;
(3) $\lambda Z_{i}⊕\lambda Z_{j}=\lambda \left(Z_{i}⊕Z_{j}\right)$;
(4) $\lambda_{1}Z_{i}⊕\lambda_{2}Z_{i}=\left(\lambda_{1}+\lambda_{2}\right)Z_{i}$;
(5) $Z_{i}⊗Z_{j}=Z_{j}⊗Z_{i}$;
(6) $\left(Z_{i}⊗Z_{j}\right)⊗Z_{k}=Z_{i}⊗\left(Z_{j}⊗Z_{k}\right)$;
(7) $Z_{i}^{\lambda }⊗Z_{j}^{\lambda }={\left(Z_{i}⊗Z_{j}\right)}^{\lambda }$;
(8) $Z_{i}^{\lambda_{1}}⊕Z_{i}^{\lambda_{2}}=Z_{i}^{\left(\lambda_{1}+\lambda_{2}\right)}$.

### 4.2. Power Weighted Average Operator with Z-IVLNS-TTCs

In solving real-life problems, an important step is to synthesize all the evaluation information. In this case, a Z-IVLNS-TTC power-weighted average operator (Z-IVLNS-TTCPWAO) is proposed.

**Definition 13.**  *Assume that Z-IVLNS-TTCs $\left\{Z_{i},\left(i=1,2,\cdots ,n\right)\right\}$, the corresponding weight vector is $W=\{w_{1},w_{2},\cdots ,w_{n}\}$. The integrated operator can be derived.*

(9) $$\begin{array}{c} Z\;\;-\;IVLNS\;\;-\;TTCPWAO\left(Z_{1},Z_{2},\cdots ,Z_{n}\right)\;\;=\;\;{\left(\sum_{i=1}^{n}\gamma_{i}{\underline{Ex_{i}}}^{\lambda }\right)}^{\frac{1}{\lambda }-1}\sqrt{\sum_{i=1}^{n}\gamma_{i}{\underline{Ex_{i}}}^{2\lambda -2}{\underline{He_{i}}}^{2}},{\left(\sum_{i=1}^{n}\gamma_{i}{\underline{Ex_{i}}}^{\lambda }\right)}^{\frac{1}{\lambda }-1}\sqrt{\sum_{i=1}^{n}\gamma_{i}{\underline{Ex_{i}}}^{2\lambda -2}{\underline{En_{i}}}^{2}},[{\left(\sum_{i=1}^{n}\gamma_{i}{\underline{Ex_{i}}}^{\lambda }\right)}^{\frac{1}{\lambda }},\\ {\left(\sum_{i=1}^{n}\gamma_{i}{\bar{Ex_{i}}}^{\lambda }\right)}^{\frac{1}{\lambda }}],{\left(\sum_{i=1}^{n}\gamma_{i}{\bar{Ex_{i}}}^{\lambda }\right)}^{\frac{1}{\lambda }-1}\sqrt{\sum_{i=1}^{n}\gamma_{i}{\bar{Ex_{i}}}^{2\lambda -2}{\bar{En_{i}}}^{2}},{\left(\sum_{i=1}^{n}\gamma_{i}{\bar{Ex_{i}}}^{\lambda }\right)}^{\frac{1}{\lambda }-1}\sqrt{\sum_{i=1}^{n}\gamma_{i}{\bar{Ex_{i}}}^{2\lambda -2}{\bar{He_{i}}}^{2}},\\ {\left(\frac{\sum_{i=1}^{n}\gamma_{i}Ex_{i}^{\lambda }{\underline{ex_{i}}}^{\lambda }}{\sum_{i=1}^{n}\gamma_{i}Ex_{i}^{\lambda }}\right)}^{\frac{1}{\lambda }-1}\sqrt{\frac{\sum_{i=1}^{n}\gamma_{i}Ex_{i}^{\lambda }{\underline{ex_{i}}}^{2\lambda -2}{\underline{he_{i}}}^{2}}{\sum_{i=1}^{n}\gamma_{i}Ex_{i}^{\lambda }}},{\left(\frac{\sum_{i=1}^{n}\gamma_{i}Ex_{i}^{\lambda }{\underline{ex_{i}}}^{\lambda }}{\sum_{i=1}^{n}\gamma_{i}Ex_{i}^{\lambda }}\right)}^{\frac{1}{\lambda }-1}\sqrt{\frac{\sum_{i=1}^{n}\gamma_{i}Ex_{i}^{\lambda }{\underline{ex_{i}}}^{2\lambda -2}{\underline{en_{i}}}^{2}}{\sum_{i=1}^{n}\gamma_{i}Ex_{i}^{\lambda }}},[{\left(\frac{\sum_{i=1}^{n}\gamma_{i}Ex_{i}^{\lambda }{\underline{ex_{i}}}^{\lambda }}{\sum_{i=1}^{n}\gamma_{i}Ex_{i}^{\lambda }}\right)}^{\frac{1}{\lambda }},\\ {\left(\frac{\sum_{i=1}^{n}\gamma_{i}Ex_{i}^{\lambda }{\bar{ex_{i}}}^{\lambda }}{\sum_{i=1}^{n}\gamma_{i}Ex_{i}^{\lambda }}\right)}^{\frac{1}{\lambda }}],{\left(\frac{\sum_{i=1}^{n}\gamma_{i}Ex_{i}^{\lambda }{\bar{ex_{i}}}^{\lambda }}{\sum_{i=1}^{n}\gamma_{i}Ex_{i}^{\lambda }}\right)}^{\frac{1}{\lambda }-1}\sqrt{\frac{\sum_{i=1}^{n}\gamma_{i}Ex_{i}^{\lambda }{\bar{ex_{i}}}^{2\lambda -2}{\bar{en_{i}}}^{2}}{\sum_{i=1}^{n}\gamma_{i}Ex_{i}^{\lambda }}},{\left(\frac{\sum_{i=1}^{n}\gamma_{i}Ex_{i}^{\lambda }{\bar{ex_{i}}}^{\lambda }}{\sum_{i=1}^{n}\gamma_{i}Ex_{i}^{\lambda }}\right)}^{\frac{1}{\lambda }-1}\sqrt{\frac{\sum_{i=1}^{n}\gamma_{i}Ex_{i}^{\lambda }{\bar{ex_{i}}}^{2\lambda -2}{\bar{he_{i}}}^{2}}{\sum_{i=1}^{n}\gamma_{i}Ex_{i}^{\lambda }}} \end{array}$$

where $\gamma_{i}=\frac{w_{i}\left(1+T\left(Z_{i}\right)\right)Z_{n}^{\lambda }}{\sum_{i=1}^{n}w_{i}\left(1+T\left(Z_{i}\right)\right)}$, $Ex_{i}=\frac{1}{2}\left(\underline{Ex_{i}}+\bar{Ex_{i}}\right)$, and $ex_{i}=\frac{1}{2}\left(\underline{ex_{i}}+\bar{ex_{i}}\right)$. $T\left(Z_{i}\right)=\sum_{j=1,j\neq i}^{n}w_{j}Sup\left(Z_{i},Z_{j}\right)$ is the comprehensive weighted support degree of $Z_{i}$ from $Z_{j}$; $Sup(Z_{i},Z_{j})$ denotes the support degree of $Z_{i}$ from $Z_{j}$.

The support degree satisfies the following properties:

(1) $Sup\left(Z_{i},Z_{j}\right)\in \left[0,1\right]$;
(2) $Sup\left(Z_{i},Z_{j}\right)=Sup\left(Z_{j},Z_{i}\right)$;
(3) $Sup\left(Z_{i},Z_{j}\right)\geq Sup\left(Z_{k},Z_{l}\right)$, if $d\left(Z_{i},Z_{j}\right)\leq d\left(Z_{k},Z_{l}\right)$, where $d(Z_{i},Z_{j})$ indicates the distance between $Z_{i}$ and $Z_{j}$; let $Sup\left(Z_{i},Z_{j}\right)=1-\frac{d(Z_{i},Z_{j})}{\sum_{i=1}^{n-1}\sum_{j=i+1}^{n}d\left(Z_{i},Z_{j}\right)}$.

**Definition 14.**  *There have been studies indicating that the key point with regard to selecting the weight vector is to maximize the information of each criterion to avoid excessive information loss.For Z-IVLNS-TTCs, the following formulas can be established.*

(10) $$\max:\left\{\begin{array}{c} f_{1}\left(W\right)={\left(\sum_{i=1}^{n}\gamma_{i}{\underline{Ex_{i}}}^{\lambda }\right)}^{\frac{1}{\lambda }};\;\;\;\;\;\;\\ f_{2}\left(W\right)={\left(\sum_{i=1}^{n}\gamma_{i}{\bar{Ex_{i}}}^{\lambda }\right)}^{\frac{1}{\lambda }};\;\;\;\;\;\;\\ f_{3}\left(W\right)={\left(\sum_{i=1}^{n}\gamma_{i}Ex_{i}^{\gamma }{\underline{ex_{i}}}^{\gamma }/\sum_{i=1}^{n}\gamma_{i}Ex_{i}^{\gamma }\right)}^{\frac{1}{\lambda }};\\ f_{4}\left(W\right)={\left(\sum_{i=1}^{n}\gamma_{i}Ex_{i}^{\gamma }{\bar{ex_{i}}}^{\gamma }/\sum_{i=1}^{n}\gamma_{i}Ex_{i}^{\gamma }\right)}^{\frac{1}{\lambda }}. \end{array}\right.$$

(11) $$\min:\left\{\begin{array}{c} g_{1}\left(W\right)={\left(\sum_{i=1}^{n}\gamma_{i}{\underline{Ex_{i}}}^{\lambda }\right)}^{\frac{1}{\lambda }-1}\sqrt{\sum_{i=1}^{n}\gamma_{i}{\underline{Ex_{i}}}^{2\lambda -2}{\underline{He_{i}}}^{2}};\\ g_{2}\left(W\right)={\left(\sum_{i=1}^{n}\gamma_{i}{\underline{Ex_{i}}}^{\lambda }\right)}^{\frac{1}{\lambda }-1}\sqrt{\sum_{i=1}^{n}\gamma_{i}{\underline{Ex_{i}}}^{2\lambda -2}{\underline{En_{i}}}^{2}};\\ g_{3}\left(W\right)={\left(\sum_{i=1}^{n}\gamma_{i}{\bar{Ex_{i}}}^{\lambda }\right)}^{\frac{1}{\lambda }-1}\sqrt{\sum_{i=1}^{n}\gamma_{i}{\bar{Ex_{i}}}^{2\lambda -2}{\bar{En_{i}}}^{2}};\\ g_{4}\left(W\right)={\left(\sum_{i=1}^{n}\gamma_{i}{\bar{Ex_{i}}}^{\lambda }\right)}^{\frac{1}{\lambda }-1}\sqrt{\sum_{i=1}^{n}\gamma_{i}{\bar{Ex_{i}}}^{2\lambda -2}{\bar{He_{i}}}^{2}};\\ g_{5}\left(W\right)={\left(\frac{\sum_{i=1}^{n}\gamma_{i}Ex_{i}^{\gamma }ex_{i}^{\gamma }}{\sum_{i=1}^{n}\gamma_{i}Ex_{i}^{\gamma }}\right)}^{\frac{1}{\lambda }-1}\sqrt{\frac{\sum_{i=1}^{n}\gamma_{i}Ex_{i}^{\gamma }ex_{i}^{2\gamma -2}{\underline{he_{i}}}^{2}}{\sum_{i=1}^{n}\gamma_{i}Ex_{i}^{\gamma }}};\;\\ g_{6}\left(W\right)={\left(\frac{\sum_{i=1}^{n}\gamma_{i}Ex_{i}^{\gamma }ex_{i}^{\gamma }}{\sum_{i=1}^{n}\gamma_{i}Ex_{i}^{\gamma }}\right)}^{\frac{1}{\lambda }-1}\sqrt{\frac{\sum_{i=1}^{n}\gamma_{i}Ex_{i}^{\gamma }ex_{i}^{2\gamma -2}{\underline{en_{i}}}^{2}}{\sum_{i=1}^{n}\gamma_{i}Ex_{i}^{\gamma }}};\;\\ g_{7}\left(W\right)={\left(\frac{\sum_{i=1}^{n}\gamma_{i}Ex_{i}^{\gamma }ex_{i}^{\gamma }}{\sum_{i=1}^{n}\gamma_{i}Ex_{i}^{\gamma }}\right)}^{\frac{1}{\lambda }-1}\sqrt{\frac{\sum_{i=1}^{n}\gamma_{i}Ex_{i}^{\gamma }ex_{i}^{2\gamma -2}{\bar{en_{i}}}^{2}}{\sum_{i=1}^{n}\gamma_{i}Ex_{i}^{\gamma }}};\;\\ g_{8}\left(W\right)={\left(\frac{\sum_{i=1}^{n}\gamma_{i}Ex_{i}^{\gamma }ex_{i}^{\gamma }}{\sum_{i=1}^{n}\gamma_{i}Ex_{i}^{\gamma }}\right)}^{\frac{1}{\lambda }-1}\sqrt{\frac{\sum_{i=1}^{n}\gamma_{i}Ex_{i}^{\gamma }ex_{i}^{2\gamma -2}{\bar{he_{i}}}^{2}}{\sum_{i=1}^{n}\gamma_{i}Ex_{i}^{\gamma }}}.\; \end{array}\right.$$

The constraints are as follows:

(12) $$s.t.\left\{\begin{array}{c} \sum_{i=1}^{n}w_{i}=1;\;\;\\ 0\leq w_{i}\leq 1.\;\; \end{array}\right.$$

To address the problem, we adopt the following approach [34].

(1) Construct the objective function F(W).

(13) $$\begin{array}{c} F\left(W\right)=\min(-f_{1}\left(W\right)-f_{2}\left(W\right)-f_{3}\left(W\right)-f_{4}\left(W\right)+g_{1}\left(W\right)+g_{2}\left(W\right)+g_{3}\left(W\right)+g_{4}\left(W\right)+g_{5}\left(W\right)+g_{6}\left(W\right)+g_{7}\left(W\right)+g_{8}\left(W\right)) \end{array}$$

(14) $$\begin{array}{c} s.t\;\left\{\begin{array}{c} G_{k}\left(W\right)=-w_{j}\leq 0,\;\left(k=1,\;2,\cdots ,\;n\right)\\ G_{k}\left(W\right)=w_{j}-1\leq 0,\;\left(k=n+1,\cdots ,\;2n\right)\\ H\left(W\right)=w_{1}+w_{2}+\cdots +w_{n}-1=0 \end{array}\right. \end{array}$$

(2) Add the slack variable vector $Z=\{z_{1},\cdots ,z_{2n}\}$ to transform the inequality constraints into equality constraints.
  (15) $$\begin{array}{c} s.t\;\left\{\begin{array}{c} {\tilde{G}}_{k}\left(W,\;Z\right)=-w_{j}+z_{k}^{2}=0,\;\left(k=1,\;2,\cdots ,\;n\right)\\ {\tilde{G}}_{k}\left(W,\;Z\right)=w_{j}-1+z_{k}^{2}=0,\;\left(k=n+1,\cdots ,\;2n\right)\\ \tilde{H}\left(W\right)=H\left(W\right)=w_{1}+w_{2}+\cdots +w_{n}-1=0 \end{array}\right. \end{array}$$

(3) Transform the problem into unconstrained minimization problem with Lagrange multiplier method.
  (16) $$\begin{array}{c} \min F\left(W,Z,\mu ,\lambda ,M\right)=F\left(W\right)+\sum_{j=1}^{n}\mu_{j}{\tilde{G}}_{k}\left(W,Z\right)+\sum_{j=n+1}^{2n}\mu_{j}{\tilde{G}}_{k}\left(W,Z\right)+\lambda \tilde{H}\left(W\right)\\ +\frac{1}{2}n\left\{\sum_{j=1}^{n}{\left[{\tilde{G}}_{k}\left(W,Z\right)\right]}^{2}+\sum_{j=n+1}^{2n}{\left[{\tilde{G}}_{k}\left(W,Z\right)\right]}^{2}+{\left[\tilde{H}\left(W\right)\right]}^{2}\right\} \end{array}$$
  where $\mu_{j}$, λ, and *M* indicate the Lagrange multipliers.

(4) To address the problem, simplex evolutionary, an available optimization method, is employed. The calculation process is shown in [35].

### 4.3. Distance Calculation Method Between Z-IVLNS-TTCs

In (9), the distance measure $d(Z_{i},Z_{j})$ is unknown. For this purpose, a new distance calculation method is proposed.

**Definition 15.**  *Let $Z_{i}=\left(\left(\underline{He_{i}},\underline{En_{i}},\left[\underline{Ex_{i}},\bar{Ex_{i}}\right],\bar{En_{i}},\bar{He_{i}}\right),\left(\underline{he_{i}},\underline{en_{i}},\left[\underline{ex_{i}},\bar{ex_{i}}\right],\bar{en_{i}},\bar{he_{i}}\right)\right)$ and $Z_{j}=\left(\left(\underline{He_{j}},\underline{En_{j}},\left[\underline{Ex_{j}},\bar{Ex_{j}}\right],\bar{En_{j}},\bar{He_{j}}\right),\left(\underline{he_{j}},\underline{en_{j}},\left[\underline{ex_{j}},\bar{ex_{j}}\right],\bar{en_{j}},\bar{he_{j}}\right)\right)$, the p-norm distance of $Z_{i}$ from $Z_{j}$ is:*

(17) $$\begin{array}{c} D_{1}\left(Z_{i},Z_{j}\right)\;\;=[\;\frac{1}{6}\;[{\left(\frac{|\underline{He_{i}}-\underline{He_{j}}|}{|X_{\max}-X_{\min}|}\right)}^{p}\;\;+\;\;{\left(\;\frac{|\underline{En_{i}}-\underline{En_{j}}|}{|X_{\max}-X_{\min}|}\right)}^{p}\;\;\\ +{\left(\frac{|\underline{Ex_{i}}-\underline{Ex_{j}}|}{|X_{\max}-X_{\min}|}\;\right)}^{p}+{\left(\frac{|\bar{Ex_{i}}-\bar{Ex_{j}}|}{|X_{\max}-X_{\min}|}\right)}^{p}\;\;\;\;\;\\ +{\left(\frac{|\bar{En_{i}}-\bar{En_{j}}|}{|X_{\max}-X_{\min}|}\right)}^{p}+{\left(\frac{|\bar{He_{i}}-\bar{He_{j}}|}{|X_{\max}-X_{\min}|}\right)}^{p}]{]}^{1/p}. \end{array}$$

and

(18) $$\begin{array}{c} D_{2}\left(Z_{i},Z_{j}\right)\;\;=[\;\frac{1}{6}\;[{\left(\frac{|\underline{he_{i}}-\underline{he_{j}}|}{|X_{\max}-X_{\min}|}\right)}^{p}\;\;+\;\;{\left(\;\frac{|\underline{en_{i}}-\underline{en_{j}}|}{|X_{\max}-X_{\min}|}\right)}^{p}\;\;\\ +{\left(\frac{|\underline{ex_{i}}-\underline{ex_{j}}|}{|X_{\max}-X_{\min}|}\;\right)}^{p}+{\left(\frac{|\bar{ex_{i}}-\bar{ex_{j}}|}{|X_{\max}-X_{\min}|}\right)}^{p}\;\;\;\;\;\\ +{\left(\frac{|\bar{en_{i}}-\bar{en_{j}}|}{|X_{\max}-X_{\min}|}\right)}^{p}+{\left(\frac{|\bar{he_{i}}-\bar{he_{j}}|}{|X_{\max}-X_{\min}|}\right)}^{p}]{]}^{1/p}. \end{array}$$

where (p=1,2,⋯).

The final result is defined as follows:

(19) $$\begin{array}{c} D\left(Z_{i},Z_{j}\right)=\frac{1}{2}\left[D_{1}\left(Z_{i},Z_{j}\right)+D_{2}\left(Z_{i},Z_{j}\right)\right]. \end{array}$$

What needs to be emphasized is that there are four prerequisites that the formula for calculating the distance must satisfy [36].

**Theorem 1.**  *Suppose that A, B, and C are three cloud models, if D(A,B) meets the following conditions, then it is called the generalized distance between A and B:*

(1) 0≤D(A,B)≤1;
(2) If A=B, then D(A,B)=0;
(3) D(A,B)=D(B,A);
(4) If A⊆B⊆C, then D(A,C)≥D(A,B) and D(A,C)≥D(B,C).

**Proof.**  (1) 0≤D(A,B)≤1.

Considering that

$$\left\{\underline{He_{i}},\underline{En_{i}},\underline{Ex_{i}},\bar{Ex_{i}},\bar{En_{i}},\bar{He_{i}}\right\}\in \left[X_{\min},X_{\max}\right]$$

and

$$\left\{\underline{he_{i}},\underline{en_{i}},\underline{ex_{i}},\bar{ex_{i}},\bar{en_{i}},\bar{he_{i}}\right\}\in \left[X_{\min},X_{\max}\right]$$

→

$$\begin{array}{c} 0\leq {\left(\frac{|\underline{He_{i}}-\underline{He_{j}}|}{|X_{\max}\;-\;X_{\min}|}\right)}^{p}\;\;+\;\;{\left(\;\frac{|\underline{En_{i}}-\underline{En_{j}}|}{|X_{\max}\;-\;X_{\min}|}\right)}^{p}+{\left(\frac{|\underline{Ex_{i}}-\underline{Ex_{j}}|}{|X_{\max}\;-\;X_{\min}|}\;\right)}^{p}+\;{\left(\frac{|\bar{Ex_{i}}-\bar{Ex_{j}}|}{|X_{\max}\;-\;X_{\min}|}\right)}^{p}\;\;+\;\;{\left(\frac{|\bar{En_{i}}-\bar{En_{j}}|}{|X_{\max}\;-\;X_{\min}|}\right)}^{p}\;\;+\;\;{\left(\frac{|\bar{He_{i}}-\bar{He_{j}}|}{|X_{\max}\;-\;X_{\min}|}\right)}^{p}\;\leq \;6;\;\; \end{array}$$

and

$$\begin{array}{c} 0\leq {\left(\frac{|\underline{he_{i}}-\underline{he_{j}}|}{|X_{\max}\;-\;X_{\min}|}\right)}^{p}\;+\;{\left(\frac{|\underline{en_{i}}-\underline{en_{j}}|}{|X_{\max}\;-\;X_{\min}|}\right)}^{p}\;+\;{\left(\frac{|\underline{ex_{i}}-\underline{ex_{j}}|}{|X_{\max}\;-\;X_{\min}|}\;\right)}^{p}+\;{\left(\frac{|\bar{ex_{i}}-\bar{ex_{j}}|}{|X_{\max}\;-\;X_{\min}|}\right)}^{p}\;\;+\;\;{\left(\frac{|\bar{en_{i}}-\bar{en_{j}}|}{|X_{\max}\;-\;X_{\min}|}\right)}^{p}\;\;+\;\;{\left(\frac{|\bar{he_{i}}-\bar{he_{j}}|}{|X_{\max}\;-\;X_{\min}|}\right)}^{p}]\;\leq \;6.\;\;\;\;\; \end{array}$$

Therefore, $0\leq D(Z_{i},Z_{j})\leq 1$.

In (2) and (3), the two conditions can be easily found to be achievable and we do not prove them in detail.

(4) If A⊆B⊆C, then D(A,C)≥D(A,B) and D(A,C)≥D(B,C).

If $Z_{i}\subseteq Z_{j}\subseteq Z_{k}$, then:

$\underline{He_{i}}\geq \underline{He_{j}}\geq \underline{He_{k}}$, $\underline{En_{i}}\geq \underline{En_{j}}\geq \underline{En_{k}}$, $\underline{Ex_{i}}\leq \underline{Ex_{j}}\leq \underline{Ex_{k}}$, $\bar{Ex_{i}}\leq \bar{Ex_{j}}\leq \bar{Ex_{k}}$, $\bar{En_{i}}\geq \bar{En_{j}}\geq \bar{En_{k}}$, $\bar{He_{i}}\geq \bar{He_{j}}\geq \bar{He_{k}}$, $\underline{he_{i}}\geq \underline{he_{j}}\geq \underline{he_{k}}$, $\underline{en_{i}}\geq \underline{en_{j}}\geq \underline{en_{k}}$, $\underline{ex_{i}}\leq \underline{ex_{j}}\leq \underline{ex_{k}}$, $\bar{ex_{i}}\leq \bar{ex_{j}}\leq \bar{ex_{k}}$, $\bar{en_{i}}\geq \bar{en_{j}}\geq \bar{en_{k}}$, $\bar{he_{i}}\geq \bar{he_{j}}\geq \bar{he_{k}}$.

The following inequalities can be deduced:

$-|\underline{He_{i}}-\underline{He_{j}}\left|\geq -\right|\underline{He_{i}}-\underline{He_{k}}|$, $-|\underline{En_{i}}-\underline{En_{j}}\left|\geq -\right|\underline{En_{i}}-\underline{En_{k}}|$, $-|\underline{Ex_{i}}-\underline{Ex_{j}}\left|\geq -\right|\underline{Ex_{i}}-\underline{Ex_{k}}|$, $-|\bar{Ex_{i}}-\bar{Ex_{j}}\left|\geq -\right|\bar{Ex_{i}}-\bar{Ex_{k}}|$, $-|\bar{En_{i}}-\bar{En_{j}}\left|\geq -\right|\bar{En_{i}}-\bar{En_{k}}|$, $-|\bar{He_{i}}-\bar{He_{j}}\left|\geq -\right|\bar{He_{i}}-\bar{He_{k}}|$;

And

$-|\underline{he_{i}}-\underline{he_{j}}\left|\geq -\right|\underline{he_{i}}-\underline{he_{k}}|$, $-|\underline{en_{i}}-\underline{en_{j}}\left|\geq -\right|\underline{en_{i}}-\underline{en_{k}}|$, $-|\underline{ex_{i}}-\underline{ex_{j}}\left|\geq -\right|\underline{ex_{i}}-\underline{ex_{k}}|$, $-|\bar{ex_{i}}-\bar{ex_{j}}\left|\geq -\right|\bar{ex_{i}}-\bar{ex_{k}}|$, $-|\bar{en_{i}}-\bar{en_{j}}\left|\geq -\right|\bar{en_{i}}-\bar{en_{k}}|$, $-|\bar{he_{i}}-\bar{he_{j}}\left|\geq -\right|\bar{he_{i}}-\bar{he_{k}}|$.

Thus

$D_{1}\left(Z_{i},Z_{k}\right)\geq D_{1}\left(Z_{i},Z_{j}\right)$ and $D_{2}\left(Z_{i},Z_{k}\right)\geq D_{2}\left(Z_{i},Z_{j}\right)$.

So, $D\left(Z_{i},Z_{k}\right)\geq D\left(Z_{i},Z_{j}\right)$.

Similar,

$D\left(Z_{i},Z_{k}\right)\geq D\left(Z_{j},Z_{k}\right)$. □

### 4.4. Algorithm Process

Suppose that there are *m* decision-makers $\left\{e_{1},e_{2},\cdots ,e_{m}\right\}$ with weight vector $\iota =\{\iota_{1},\iota_{2},\cdots ,\iota_{m}\}$. Meanwhile, *q* alternatives $\left\{a_{1},a_{2},\cdots ,a_{q}\right\}$ and *k* criteria $\left\{c_{1},c_{2},\cdots ,c_{k}\right\}$ with weight vector $\left\{w_{1},w_{2},\cdots ,w_{k}\right\}$ are offered. The effective domain is $\left[X_{\min},X_{\max}\right]$. The main procedures of the proposed method can be summarized as follows.

*Step 1:*  Convert the evaluation information into Z-IVLNS-TTCs.

Utilize *Definition 2* and the conversion method (4)–(8) to transform the linguistic evaluation information given by decision-makers into Z-IVLNS-TTCs $Z(\left(\underline{He},\underline{En},\left[\underline{Ex},\bar{Ex}\right],\bar{En},\bar{He}\right),\left(\underline{he},\underline{en},\left[\underline{ex},\bar{ex}\right],\bar{en},\bar{he}\right))$.

*Step 2:*  Calculate the weights of criteria.

Employ *Definition 14* to obtain the weight vector.

Decision-maker $e_{1}$:

$$\begin{array}{c} a_{1}:W_{1}^{\left(1\right)}=\left\{w_{1}^{\left(1\right)},w_{2}^{\left(1\right)},\cdots ,w_{k}^{\left(1\right)}\right\};\\ a_{2}:W_{1}^{\left(2\right)}=\left\{w_{1}^{\left(2\right)},w_{2}^{\left(2\right)},\cdots ,w_{k}^{\left(2\right)}\right\};\\ \vdots \;\;\;\;\\ a_{q}:W_{1}^{\left(q\right)}=\left\{w_{1}^{\left(q\right)},w_{2}^{\left(q\right)},\cdots ,w_{k}^{\left(q\right)}\right\}.\\ \vdots \;\;\;\;\;\;\;\;\;\;\;\; \end{array}$$

Decision-maker $e_{m}$:

$$\begin{array}{c} a_{1}:W_{m}^{\left(1\right)}=\left\{w_{1}^{\left(1\right)},w_{2}^{\left(1\right)},\cdots ,w_{k}^{\left(1\right)}\right\};\\ a_{2}:W_{m}^{\left(2\right)}=\left\{w_{1}^{\left(2\right)},w_{2}^{\left(2\right)},\cdots ,w_{k}^{\left(2\right)}\right\};\\ \vdots \;\;\;\;\\ a_{q}:W_{m}^{\left(q\right)}=\left\{w_{1}^{\left(q\right)},w_{2}^{\left(q\right)},\cdots ,w_{k}^{\left(q\right)}\right\}. \end{array}$$

*Step 3:*  Obtain the collective evaluation information.

After obtaining the weight vector of each attribute in different cases, the integrated evaluation information expressed by Z-IVLNS-TTCs can be calculated by (9).

*Step 4:*  Calculate the distances between Z-IVLNS-TTCs.

To select the optimal alternative, a beneficial solution is to learn from TOPSIS method and quantify the distances between the Z-IVLNS-TTCs revealing the evaluation information of whole alternatives and the Z-IVLNS-TTCs that denote the positive ideal solution $Z_{p}$ and negative ideal solution $Z_{n}$ with *Definition 15*, recorded as $D_{p}$ and $D_{n}$. According to (17)–(19),

$$\begin{array}{c} Z_{p}=\left(\left({\underline{He_{i}}}_{\min},{\underline{En_{i}}}_{\min},\left[X_{\max},X_{\max}\right],{\bar{En_{i}}}_{\min},{\bar{He_{i}}}_{\min}\right),\left({\underline{he_{i}}}_{\min},{\underline{en_{i}}}_{\min},\left[X_{\max},X_{\max}\right],{\bar{en_{i}}}_{\min},{\bar{he_{i}}}_{\min}\right)\right)\\ =(\left(0,0,\left[X_{\max},X_{\max}\right],0,0\right),\left(0,0,\left[X_{\max},X_{\max}\right],0,0\right));\;\;\;\;\;\;\;\; \end{array}$$

and

$$\begin{array}{c} Z_{n}=\left(\left({\underline{He_{i}}}_{\max},{\underline{En_{i}}}_{\max},\left[X_{\min},X_{\min}\right],{\bar{En_{i}}}_{\max},{\bar{He_{i}}}_{\max}\right),\left({\underline{he_{i}}}_{\max},{\underline{en_{i}}}_{\max},\left[X_{\min},X_{\min}\right],{\bar{en_{i}}}_{\max},{\bar{he_{i}}}_{\max}\right)\right)\\ =\;\;(\left(\frac{X_{\max}\;\;-\;\;X_{\min}}{9},\;\frac{X_{\max}\;\;-\;\;X_{\min}}{3},\;\left[X_{\min},X_{\min}\right],\;\frac{X_{\max}\;\;-\;\;X_{\min}}{3},\;\frac{X_{\max}\;\;-\;\;X_{\min}}{9}\right),\;\;\;\\ (\frac{X_{\max}\;\;-\;\;X_{\min}}{9},\;\frac{X_{\max}\;\;-\;\;X_{\min}}{3},\;\left[X_{\min},X_{\min}\right],\;\frac{X_{\max}\;\;-\;\;X_{\min}}{3},\;\frac{X_{\max}\;\;-\;\;X_{\min}}{9})).\;\;\; \end{array}$$

*Step 5:*  Rank all of the alternatives.

**Figure 1:**
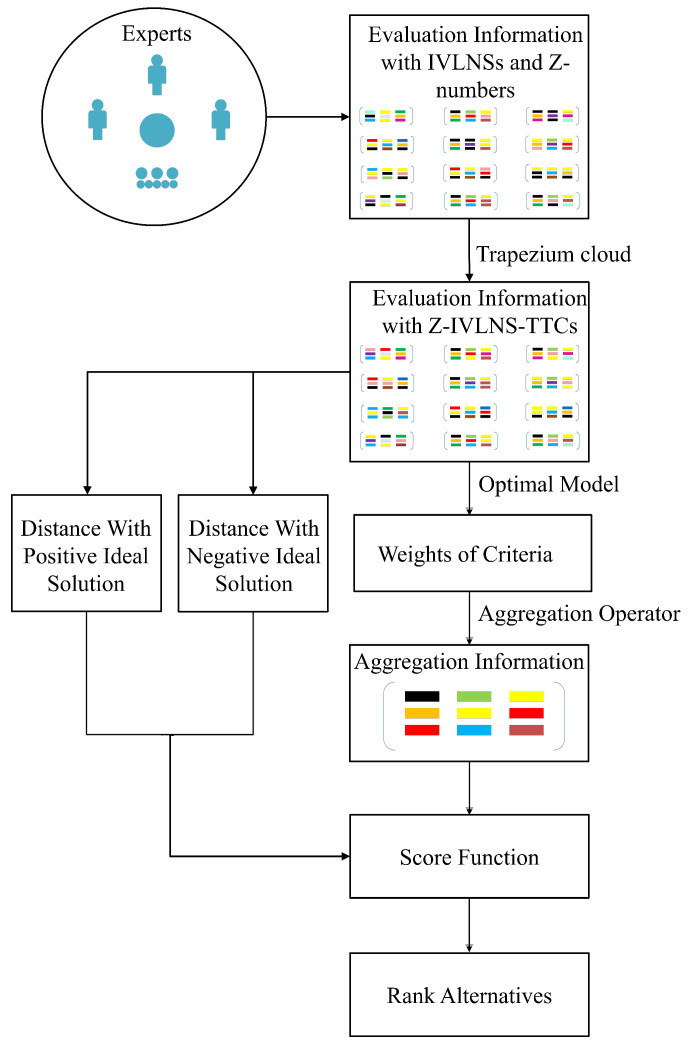
Whole algorithm process.

Utilize the formula $D=D_{n}/\left(D_{p}+D_{n}\right)$ to rank all alternatives, and then, regard the conclusion with the highest score as the final result. (See Figure 1).

## 5. Illustrative Example

Leading international aircraft manufacturers, including Boeing and Airbus, have built their own VHM based on two-way data communication systems. The main objective is to obtain the timely health status of the aircraft, i.e., a health level assessment. In view of the fact that it is convenient to describe many factors in language, the proposed method is employed. Assume that there are four states *health* $\left(a_{1}\right)$, *sub-health*$\left(a_{2}\right)$, *qualified*$\left(a_{3}\right)$, and *fault*$\left(a_{4}\right)$. The main factors are *status of sensors*$\left(c_{1}\right)$, *status of aircraft structure*$\left(c_{2}\right)$, *status of engine*$\left(c_{3}\right)$, and *status of hydraulic system*$\left(c_{4}\right)$. Obviously, it is difficult to describe the four factors with accurate values. For reference, three experts $\left\{d_{1},d_{2},d_{3}\right\}$ offer personal evaluation information with linguistic term sets $H=\{h_{0}=$ extremely poor, $h_{1}=$ very poor, $h_{2}=$ poor, $h_{3}=$ slightly poor, $h_{4}=$ fair, $h_{5}=$ slightly good, $h_{6}=$ good, $h_{7}=$ very good, $h_{8}=$ extremely good} and $S=\{s_{0}=$ extremely uncertain, $s_{1}=$ very uncertain, $s_{2}=$ uncertain, $s_{3}=$ slightly uncertain, $s_{4}=$ neutral, $s_{5}=$ slightly certain, $s_{6}=$ certain, $s_{7}=$ very certain, $s_{8}=$ extremely certain}. The weight vector of experts is $\eta_{1}=\eta_{2}=\eta_{3}=1/3$. The evaluation information is displayed in Table 1, Table 2 and Table 3.

The procedures are summarized in the following steps. Let the effective domain be U=[5,95], λ=2, and p=5.

*Step 1:*  Convert the evaluation information into Z-IVLNS-TTCs.

Z-IVLNS-TTCs expressing linguistic information can be obtained by (4)–(8), as shown in Table 4, Table 5 and Table 6.

*Step 2:*  Calculate the weights of criteria.

The weights can be acquired by using *Definition 14*, the result is shown below.

Expert 1:

$$\begin{array}{c} a_{1}:\;W_{1}^{\left(1\right)}=\left\{0.2437,0.2553,0.2319,0.2691\right\}; \end{array}$$

$$\begin{array}{c} a_{2}:\;W_{1}^{\left(2\right)}=\left\{0.2503,0.2496,0.2486,0.2515\right\}; \end{array}$$

$$\begin{array}{c} a_{3}:\;W_{1}^{\left(3\right)}=\left\{0.2440,0.2483,0.2605,0.2472\right\}; \end{array}$$

$$\begin{array}{c} a_{4}:\;W_{1}^{\left(4\right)}=\left\{0.2551,0.2508,0.2489,0.2452\right\}. \end{array}$$

Expert 2:

$$\begin{array}{c} a_{1}:\;W_{2}^{\left(1\right)}=\left\{0.2671,0.2497,0.2438,0.2394\right\}; \end{array}$$

$$\begin{array}{c} a_{2}:\;W_{2}^{\left(2\right)}=\left\{0.2589,0.2416,0.2504,0.2491\right\}; \end{array}$$

$$\begin{array}{c} a_{3}:\;W_{2}^{\left(3\right)}=\left\{0.2399,0.2508,0.2471,0.2622\right\}; \end{array}$$

$$\begin{array}{c} a_{4}:\;W_{2}^{\left(4\right)}=\left\{0.2559,0.2536,0.2412,0.2493\right\}. \end{array}$$

Expert 3:

$$\begin{array}{c} a_{1}:\;W_{3}^{\left(1\right)}=\left\{0.2557,0.2489,0.2470,0.2484\right\}; \end{array}$$

$$\begin{array}{c} a_{2}:\;W_{3}^{\left(2\right)}=\left\{0.2671,0.2447,0.2502,0.2380\right\}; \end{array}$$

$$\begin{array}{c} a_{3}:\;W_{3}^{\left(3\right)}=\left\{0.2479,0.2491,0.2611,0.2419\right\}; \end{array}$$

$$\begin{array}{c} a_{4}:\;W_{3}^{\left(4\right)}=\left\{0.2661,0.2309,0.2512,0.2518\right\}. \end{array}$$

*Step 3:*  Obtain the collective evaluation information.

Based on the weights obtained in *Step 2*, collective evaluation information can be synthesized with (9). The integrated trapezium clouds are shown in Table 7.

*Step 4:*  Calculate the distances between Z-IVLNS-TTCs.

The distances between the Z-IVLNS-TTCs expressing the evaluation information and the ideal trapezium clouds can be obtained with *Definition 15*, $Z_{p}=(\left(0,0,\left[95,95\right],0,0\right),$(0,0,[95,95],0,0)) and $Z_{n}=\left((10,30,[5,5],30,10),(10,30,[5,5],30,10)\right)$. The results are shown in Table 8.

*Step 5:*  Rank all of the alternatives.

Table 9 reveals the average distances of different alternatives, and then, the final ranking is offered.

## 6. Comparative Analysis and Discussion

To illustrate the rationality of the proposed method, a comparison is performed between the other four common methods in [29,30,31,37]. The comparison analysis is based on the same illustrative example.

### 6.1. Comparison of Ranking Results

In [29], the decision-makers’ linguistic evaluation information is transformed into quantitative data using the normal cloud model. Subsequently, a linguistic MCGDM method is introduced, which utilizes the aggregation operators of the cloud model. This approach shares similarities with the methods described in [30], which also follow a comparable line of thought to rank alternatives. However, to overcome the limitations of the normal cloud model in capturing the uncertainty and randomness of qualitative concepts, the trapezium cloud model is employed instead. In [31], Z-numbers are integrated with AIVIFSs to create a novel representation. Meanwhile, in [37], hesitant uncertain linguistic Z-numbers serve as a reliable tool for addressing MCGDM problems expressed in linguistic format.

The detailed results obtained by the methods in [29,30,31,37] are $a_{3}≻a_{4}≻a_{1}≻a_{2}$, $a_{4}≻a_{3}≻a_{1}≻a_{2}$, $a_{3}≻a_{4}≻a_{1}≻a_{2}$, and $a_{2}≻a_{1}≻a_{4}≻a_{3}$. The reasons for the difference will be discussed in the following part.

### 6.2. Further Comparison with Different Methods

In general, the four methods for comparison are also dedicated to solving linguistic MCGDM problems. The difference is that the three methods in [29,30,31] mainly make use of the cloud model, while the remaining method inclines to employ Z-numbers. The purpose of the method proposed in this paper is to enhance both the ability of the cloud model to handle linguistic evaluation information and extend Z-numbers.

In [29], linguistic single-valued evaluation information achieves the quantification by normal cloud model. As for the above illustrative example, the evaluation information is not single-valued, but interval-valued $\left[A_{i},A_{j}\right]$, which seems inapplicable for the method in [29]. An effective solution is to separate the interval into $A_{i}$ and $A_{j}$; then, convert the two single-valued pieces of information into a normal cloud model, respectively; later, integrate the two clouds into one with aggregation operators. The remaining procedures are consistent with those in [29], the ranking result is $a_{3}≻a_{4}≻a_{1}≻a_{2}$. The main causes for the disaccord can be classified into the following categories.

There is a major distinction in expressing the uncertain information. Different from the newly proposed method, the method in [29] takes advantage of single-valued linguistic evaluation information, which will inevitably create a deficiency in expressing the uncertainty and randomness of MCGDM problems. Undoubtedly, Z-IVLNS-TTCs are characteristic of a stronger potential to overcome this shortcoming effectively because of the application of interval-valued linguistic terms, membership, non-membership, and uncertainty degree. In addition, different from the trapezium cloud model, the normal cloud model employed in [29] makes use of only three parameters, the expected value Ex, the entropy En, and the hyper entropy He, to achieve the conversion from qualitative concepts to quantitative values. In such a case, the original linguistic information will suffer a certain degree of loss and distortion. Thus, the ranking result obtained in [29] is inconsistent with that of the method proposed in this paper. In terms of the weights of criteria, values are given subjectively in [29] according to the preferences of decision-makers without considering the objective facts and do not own the well-knit mathematical foundation, like in the proposed method. However, the weight vector plays a critical role in the final ranking of all alternatives; therefore, the result obtained in [29] can not be guaranteed to be as convincing as possible. For the method in [30], the reasons for the inconsistency are basically the same as above, including the deficiency in expressing qualitative concepts, information loss during the transformation between qualitative concepts and quantitative values, and non-objectivity in choosing weight vector. For the method in [31] and the proposed method, the main differences come from two aspects: (1) the expression of evaluation information; (2) the calculation of objective weights.

In [31], the information is given in the form of Z-numbers and AIVIFSs, recorded as $Z=\left(\left(x_{A},\left[\mu_{A}^{L},\mu_{A}^{U}\right],\left[\nu_{A}^{L},\nu_{A}^{U}\right]\right),\left(x_{B},\left[\mu_{B}^{L},\mu_{B}^{U}\right],\left[\nu_{B}^{L},\nu_{B}^{U}\right]\right)\right)$. In this paper, however, the uncertainty degree is considered and the expression is improved to $Z=\left(\left(\left[x_{A}^{L},x_{A}^{U}\right],\left[T_{A}^{L},T_{A}^{U}\right],\left[I_{A}^{L},I_{A}^{U}\right],\left[F_{A}^{L},F_{A}^{U}\right]\right),\left(\left[x_{B}^{L},x_{B}^{U}\right],\left[T_{B}^{L},T_{B}^{U}\right],\left[I_{B}^{L},I_{B}^{U}\right],\left[F_{B}^{L},F_{B}^{U}\right]\right)\right)$, which can further reveal the uncertain information. On the other hand, the weight vector of the criteria obtained in [31] is based on the entropy weight method (EWM). However, the solution only uses four parameters $\underline{En_{i}}$, $\bar{En_{i}}$, $\underline{en_{i}}$, $\bar{en_{i}}$ and the other parameters are ignored, which obviously leads to information loss. Additionally, the concept of Z-numbers has just been proposed for ten years and the relevant theoretical research is not very solid. By far, there is little research on the entropy of linguistic Z-numbers. In [31], the approach of obtaining the entropy of Z-numbers is not proved by any theorems. However, in this paper, the weight calculation method is derived from the perspective of MOP. The whole parameters of Z-IVLNS-TTCs are considered; meanwhile, the approach aims to avoid information loss based on the essence of weights.

When we consider solving the problem from the perspective of Z-numbers, an advisable reference is the method in [37]. First of all, the information is transformed into the cloud model; then, the power aggregation operators are applied to fuse the individual cloud with the weight vector that is calculated by the optimization method; finally, the ranking of alternatives is determined according to the extended VIKOR. The result $a_{2}≻a_{1}≻a_{4}≻a_{3}$ obtained in [37] is the most similar to $a_{2}≻a_{1}≻a_{3}≻a_{4}$ among the four methods. The main reasons are as follows: Compared with the traditional linguistic evaluation information, Z-numbers are composed of the uncertainty constraint and reliability measurement, which can guarantee the description of uncertainty and randomness. Also, the weights of the criteria are calculated by the optimization method, instead of being given subjectively. Therefore, the result obtained by the method is more persuasive and credible.

In summary, the proposed method addresses the key limitations of existing approaches by refining the expression of evaluation information, employing a robust cloud model, and using objective weight calculation methods. These enhancements ensure more accurate and reliable rankings, effectively managing the inherent uncertainties in MCGDM problems.

## 7. Conclusions

This paper introduces foundational concepts related to the cloud model, neutrosophic sets, and Z-numbers, followed by proposing aggregation operators for fusing individual information. A multicriteria group decision-making method is then presented. The paper contributes to the existing literature in five key ways. First, it introduces novel representation—Z-numbers with interval-valued linguistic neutrosophic sets—which offer a more flexible and efficient means of capturing uncertainty and randomness. Second, a new cloud model, Z-IVLNS-TTCs, is developed to better handle the quantification of linguistic evaluation information, significantly reducing information loss and distortion while addressing the computational challenges associated with Z-numbers encountered in previous studies. Third, an approach to calculating objective weights based on the unique characteristics of Z-IVLNS-TTCs is proposed using multi-objective programming (MOP). This method features a rigorous mathematical foundation and effectively addresses the subjectivity issues associated with weight assignment. Fourth, a new formula for calculating the distances between Z-IVLNS-TTCs is derived using the p-norm, providing a robust ranking approach. Finally, the proposed method demonstrates flexibility, with ranking results that adjust according to parameter changes. The effectiveness of the method is validated through comprehensive comparative analysis against four other approaches.

While the proposed method addresses several shortcomings of previous studies, it also has limitations that point to future research directions. Specifically, the objective weight calculation approach, based on MOP, may yield a locally minimal solution rather than the global optimum. Future research will focus on expanding this approach through extensive data analysis to address these limitations. Also, the evaluation information is given based on the experience of experts. In the future, we will try using artificial intelligence algorithms to derive the objective data.

## Figures and Tables

**Table 1:** Evaluation information of $d_{1}$.

	$c_{1}$
$a_{1}$	$(\left(\left[h_{3},h_{5}\right],\left[0.6,0.65\right],\left[0.2,0.3\right],\left[0.2,0.24\right]\right),\left(\left[s_{1},s_{2}\right]\left[0.23,0.4\right],\left[0.12,0.13\right],\left[0.12,0.13\right]\right)$
$a_{2}$	$(\left(\left[h_{4},h_{6}\right],\left[0.6,0.69\right],\left[0.3,0.33\right],\left[0.21,0.24\right]\right),\left(\left[s_{3},s_{5}\right]\left[0.85,0.91\right],\left[0.02,0.04\right],\left[0.1,0.15\right]\right)$
$a_{3}$	$(\left(\left[h_{4},h_{7}\right],\left[0.42,0.75\right],\left[0.23,0.31\right],\left[0.03,0.04\right]\right),\left(\left[s_{4},s_{6}\right]\left[0.74,0.78\right],\left[0.21,0.24\right],\left[0.14,0.15\right]\right)$
$a_{4}$	$(\left(\left[h_{4},h_{6}\right],\left[0.6,0.72\right],\left[0.2,0.34\right],\left[0.1,0.2\right]\right),\left(\left[s_{3},s_{4}\right]\left[0.61,0.71\right],\left[0.25,0.35\right],\left[0.3,0.35\right]\right)$
	$c_{2}$
$a_{1}$	$(\left(\left[h_{2},h_{4}\right],\left[0.69,0.74\right],\left[0.23,0.34\right],\left[0.32,0.35\right]\right),\left(\left[s_{3},s_{4}\right]\left[0.78,0.84\right],\left[0.1,0.13\right],\left[0.04,0.06\right]\right)$
$a_{2}$	$(\left(\left[h_{1},h_{4}\right],\left[0.88,0.92\right],\left[0.02,0.06\right],\left[0.1,0.11\right]\right),\left(\left[s_{4},s_{6}\right]\left[0.8,0.9\right],\left[0,0.01\right],\left[0.01,0.03\right]\right)$
$a_{3}$	$(\left(\left[h_{2},h_{5}\right],\left[0.78,0.87\right],\left[0.12,0.16\right],\left[0.2,0.23\right]\right),\left(\left[s_{5},s_{6}\right]\left[0.87,0.9\right],\left[0.22,0.3\right],\left[0.13,0.18\right]\right)$
$a_{4}$	$(\left(\left[h_{4},h_{5}\right],\left[0.74,0.78\right],\left[0.2,0.3\right],\left[0.2,0.4\right]\right),\left(\left[s_{4},s_{6}\right]\left[0.8,0.89\right],\left[0.21,0.33\right],\left[0.1,0.19\right]\right)$
	$c_{3}$
$a_{1}$	$(\left(\left[h_{3},h_{5}\right],\left[0.7,0.74\right],\left[0.04,0.1\right],\left[0.3,0.34\right]\right),\left(\left[s_{4},s_{6}\right]\left[0.7,0.8\right],\left[0.2,0.23\right],\left[0.15,0.17\right]\right)$
$a_{2}$	$(\left(\left[h_{3},h_{5}\right],\left[0.81,0.9\right],\left[0.13,0.16\right],\left[0.09,0.12\right]\right),\left(\left[s_{3},s_{4}\right]\left[0.9,0.98\right],\left[0.09,0.14\right],\left[0.1,0.18\right]\right)$
$a_{3}$	$(\left(\left[h_{2},h_{5}\right],\left[0.77,0.89\right],\left[0.03,0.11\right],\left[0.19,0.2\right]\right),\left(\left[s_{2},s_{3}\right]\left[0.93,0.94\right],\left[0.02,0.03\right],\left[0.08,0.12\right]\right)$
$a_{4}$	$(\left(\left[h_{2},h_{3}\right],\left[0.7,0.79\right],\left[0.23,0.4\right],\left[0.09,0.2\right]\right),\left(\left[s_{3},s_{5}\right]\left[0.9,0.94\right],\left[0.12,0.13\right],\left[0.1,0.12\right]\right)$
	$c_{4}$
$a_{1}$	$(\left(\left[h_{3},h_{5}\right],\left[0.6,0.65\right],\left[0.2,0.3\right],\left[0.2,0.24\right]\right),\left(\left[s_{1},s_{2}\right]\left[0.23,0.4\right],\left[0.12,0.13\right],\left[0.12,0.13\right]\right)$
$a_{2}$	$(\left(\left[h_{1},h_{5}\right],\left[0.78,0.8\right],\left[0.22,0.24\right],\left[0.09,0.15\right]\right),\left(\left[s_{5},s_{6}\right]\left[0.88,0.9\right],\left[0.3,0.32\right],\left[0.13,0.18\right]\right)$
$a_{3}$	$(\left(\left[h_{3},h_{6}\right],\left[0.74,0.79\right],\left[0.12,0.22\right],\left[0.23,0.24\right]\right),\left(\left[s_{5},s_{6}\right]\left[0.79,0.89\right],\left[0.31,0.35\right],\left[0.02,0.03\right]\right)$
$a_{4}$	$(\left(\left[h_{2},h_{5}\right],\left[0.93,0.98\right],\left[0.01,0.02\right],\left[0.05,0.09\right]\right),\left(\left[s_{5},s_{6}\right]\left[0.79,0.85\right],\left[0.3,0.36\right],\left[0.12,0.13\right]\right)$

**Table 2:** Evaluation information of $d_{2}$.

	$c_{1}$
$a_{1}$	$(\left(\left[h_{1},h_{5}\right],\left[0.86,0.87\right],\left[0.12,0.14\right],\left[0.02,0.11\right]\right),\left(\left[s_{2},s_{5}\right]\left[0.57,0.6\right],\left[0.25,0.29\right],\left[0.20,0.34\right]\right)$
$a_{2}$	$(\left(\left[h_{2},h_{5}\right],\left[0.86,0.9\right],\left[0,0.1\right],\left[0.12,0.13\right]\right),\left(\left[s_{3},s_{5}\right]\left[0.61,0.66\right],\left[0.34,0.45\right],\left[0.23,0.32\right]\right)$
$a_{3}$	$(\left(\left[h_{3},h_{6}\right],\left[0.67,0.75\right],\left[0.21,0.31\right],\left[0.25,0.34\right]\right),\left(\left[s_{4},s_{6}\right]\left[0.69,0.78\right],\left[0.2,0.24\right],\left[0.14,0.15\right]\right)$
$a_{4}$	$(\left(\left[h_{4},h_{5}\right],\left[0.6,0.67\right],\left[0.2,0.3\right],\left[0.2,0.24\right]\right),\left(\left[s_{1},s_{2}\right]\left[0.23,0.4\right],\left[0.12,0.13\right],\left[0.12,0.13\right]\right)$
	$c_{2}$
$a_{1}$	$(\left(\left[h_{3},h_{4}\right],\left[0.58,0.93\right],\left[0.23,0.33\right],\left[0.04,0.2\right]\right),\left(\left[s_{1},s_{4}\right]\left[0.8,0.85\right],\left[0.13,0.21\right],\left[0.05,0.14\right]\right)$
$a_{2}$	$(\left(\left[h_{1},h_{4}\right],\left[0.78,0.93\right],\left[0.22,0.4\right],\left[0.1,0.24\right]\right),\left(\left[s_{3},s_{4}\right]\left[0.83,0.85\right],\left[0.11,0.15\right],\left[0.35,0.44\right]\right)$
$a_{3}$	$(\left(\left[h_{1},h_{5}\right],\left[0.67,0.87\right],\left[0.15,0.17\right],\left[0.21,0.3\right]\right),\left(\left[s_{3},s_{5}\right]\left[0.8,0.98\right],\left[0.04,0.11\right],\left[0.1,0.15\right]\right)$
$a_{4}$	$(\left(\left[h_{2},h_{4}\right],\left[0.71,0.74\right],\left[0.23,0.34\right],\left[0.32,0.35\right]\right),\left(\left[s_{3},s_{6}\right]\left[0.8,0.84\right],\left[0.11,0.14\right],\left[0.07,0.09\right]\right)$
	$c_{3}$
$a_{1}$	$(\left(\left[h_{3},h_{6}\right],\left[0.7,0.74\right],\left[0.1,0.15\right],\left[0.1,0.14\right]\right),\left(\left[s_{2},s_{5}\right]\left[0.89,0.92\right],\left[0.03,0.09\right],\left[0.1,0.13\right]\right)$
$a_{2}$	$(\left(\left[h_{3},h_{7}\right],\left[0.71,0.75\right],\left[0.31,0.45\right],\left[0.01,0.09\right]\right),\left(\left[s_{1},s_{5}\right]\left[0.61,0.69\right],\left[0.1,0.19\right],\left[0.2,0.23\right]\right)$
$a_{3}$	$(\left(\left[h_{2},h_{4}\right],\left[0.74,0.8\right],\left[0.13,0.17\right],\left[0.29,0.3\right]\right),\left(\left[s_{2},s_{5}\right]\left[0.92,0.96\right],\left[0.12,0.13\right],\left[0.18,0.2\right]\right)$
$a_{4}$	$(\left(\left[h_{2},h_{3}\right],\left[0.72,0.76\right],\left[0.08,0.12\right],\left[0.23,0.34\right]\right),\left(\left[s_{2},s_{5}\right]\left[0.77,0.8\right],\left[0.12,0.3\right],\left[0.12,0.17\right]\right)$
	$c_{4}$
$a_{1}$	$(\left(\left[h_{2},h_{6}\right],\left[0.46,0.55\right],\left[0.43,0.46\right],\left[0.22,0.24\right]\right),(\left[s_{2},s_{4}\right]\left[0.33,0.45\right],\left[0.2,0.25,\left[0.02,0.13\right]\right)$
$a_{2}$	$(\left(\left[h_{2},h_{4}\right],\left[0.6,0.7\right],\left[0.1,0.12\right],\left[0.2,0.23\right]\right),(\left[s_{2},s_{4}\right]\left[0.33,0.44\right],\left[0.15,0.25,\left[0.02,0.13\right]\right)$
$a_{3}$	$(\left(\left[h_{2},h_{6}\right],\left[0.74,0.79\right],\left[0.12,0.26\right],\left[0.25,0.29\right]\right),\left(\left[s_{3},s_{4}\right]\left[0.9,0.93\right],\left[0.01,0.1\right],\left[0.02,0.03\right]\right)$
$a_{4}$	$(\left(\left[h_{3},h_{6}\right],\left[0.56,0.65\right],\left[0.12,0.13\right],\left[0.22,0.24\right]\right),\left(\left[s_{1},s_{5}\right]\left[0.2,0.4\right],\left[0.12,0.13\right],\left[0.12,0.13\right]\right)$

**Table 3:** Evaluation information of $d_{3}$.

	$c_{1}$
$a_{1}$	$(\left(\left[h_{2},h_{5}\right],\left[0.8,0.87\right],\left[0.1,0.2\right],\left[0.12,0.21\right]\right),\left(\left[s_{2},s_{6}\right]\left[0.7,0.76\right],\left[0.11,0.2\right],\left[0.05,0.09\right]\right)$
$a_{2}$	$(\left(\left[h_{3},h_{5}\right],\left[0.8,0.91\right],\left[0.03,0.09\right],\left[0.22,0.33\right]\right),\left(\left[s_{2},s_{5}\right]\left[0.6,0.64\right],\left[0.3,0.35\right],\left[0.24,0.29\right]\right)$
$a_{3}$	$(\left(\left[h_{3},h_{5}\right],\left[0.67,0.75\right],\left[0.21,0.31\right],\left[0.25,0.34\right]\right),\left(\left[s_{4},s_{6}\right]\left[0.69,0.78\right],\left[0.2,0.24\right],\left[0.14,0.15\right]\right)$
$a_{4}$	$(\left(\left[h_{4},h_{6}\right],\left[0.6,0.72\right],\left[0.2,0.34\right],\left[0.1,0.2\right]\right),\left(\left[s_{3},s_{4}\right]\left[0.61,0.71\right],\left[0.25,0.35\right],\left[0.3,0.35\right]\right)$
	$c_{2}$
$a_{1}$	$(\left(\left[h_{1},h_{4}\right],\left[0.8,0.9\right],\left[0.11,0.13\right],\left[0.09,0.2\right]\right),\left(\left[s_{1},s_{3}\right]\left[0.9,0.95\right],\left[0.08,0.1\right],\left[0.01,0.03\right]\right)$
$a_{2}$	$(\left(\left[h_{1},h_{4}\right],\left[0.8,0.93\right],\left[0.27,0.34\right],\left[0.1,0.24\right]\right),\left(\left[s_{1},s_{4}\right]\left[0.8,0.9\right],\left[0.01,0.1\right],\left[0.11,0.14\right]\right)$
$a_{3}$	$(\left(\left[h_{1},h_{5}\right],\left[0.67,0.87\right],\left[0.15,0.17\right],\left[0.21,0.3\right]\right),\left(\left[s_{3},s_{4}\right]\left[0.8,0.98\right],\left[0.04,0.11\right],\left[0.1,0.15\right]\right)$
$a_{4}$	$(\left(\left[h_{4},h_{6}\right],\left[0.74,0.78\right],\left[0.2,0.3\right],\left[0.2,0.4\right]\right),\left(\left[s_{4},s_{6}\right]\left[0.8,0.89\right],\left[0.21,0.33\right],\left[0.1,0.19\right]\right)$
	$c_{3}$
$a_{1}$	$(\left(\left[h_{2},h_{6}\right],\left[0.67,0.73\right],\left[0.11,0.13\right],\left[0.2,0.4\right]\right),\left(\left[s_{1},s_{5}\right]\left[0.9,0.92\right],\left[0.13,0.16\right],\left[0.01,0.13\right]\right)$
$a_{2}$	$(\left(\left[h_{2},h_{5}\right],\left[0.7,0.75\right],\left[0.1,0.11\right],\left[0.01,0.2\right]\right),\left(\left[s_{2},s_{5}\right]\left[0.41,0.5\right],\left[0.4,0.43\right],\left[0.2,0.27\right]\right)$
$a_{3}$	$(\left(\left[h_{2},h_{4}\right],\left[0.74,0.8\right],\left[0.13,0.17\right],\left[0.29,0.3\right]\right),\left(\left[s_{2},s_{5}\right]\left[0.92,0.96\right],\left[0.12,0.13\right],\left[0.18,0.2\right]\right)$
$a_{4}$	$(\left(\left[h_{1},h_{2}\right],\left[0.7,0.79\right],\left[0.23,0.4\right],\left[0.09,0.2\right]\right),\left(\left[s_{3},s_{5}\right]\left[0.9,0.94\right],\left[0.12,0.13\right],\left[0.1,0.12\right]\right)$
	$c_{4}$
$a_{1}$	$(\left(\left[h_{3},h_{4}\right],\left[0.6,0.75\right],\left[0.43,0.46\right],\left[0.22,0.24\right]\right),(\left[s_{2},s_{5}\right]\left[0.3,0.45\right],\left[0.12,0.25,\left[0.02,0.13\right]\right)$
$a_{2}$	$(\left(\left[h_{2},h_{6}\right],\left[0.64,0.7\right],\left[0.11,0.12\right],\left[0.21,0.23\right]\right),(\left[s_{1},s_{4}\right]\left[0.3,0.34\right],\left[0.15,0.25,\left[0.02,0.13\right]\right)$
$a_{3}$	$(\left(\left[h_{2},h_{5}\right],\left[0.74,0.79\right],\left[0.12,0.26\right],\left[0.25,0.29\right]\right),\left(\left[s_{3},s_{4}\right]\left[0.9,0.93\right],\left[0.01,0.1\right],\left[0.02,0.03\right]\right)$
$a_{4}$	$(\left(\left[h_{2},h_{4}\right],\left[0.93,0.98\right],\left[0.01,0.02\right],\left[0.05,0.09\right]\right),\left(\left[s_{5},s_{6}\right]\left[0.79,0.85\right],\left[0.3,0.36\right],\left[0.12,0.13\right]\right)$

**Table 4:** Z-IVLNS-TTCs evaluation information derived from $d_{1}$.

	$c_{1}$
$a_{1}$	((1.2274, 3.8355, [56.656, 73.094], 3.8355, 1.2274), (0, 6.7065, [25.9069, 40.2779], 3.4217, 3.3525))
$a_{2}$	((1.5681, 2.6974, [63.8750, 83.2965], 5.2870, 0.7049), (1.4071, 4.3972, [64.9525, 83.7975], 4.3972, 1.4071))
$a_{3}$	((1.4423, 2.4810, [58.7500, 91.2014], 6.8079,0), (1.6448, 2.8294, [67, 87.3716], 5.5456, 0.7394))
$a_{4}$	((1.5589, 2.6816, [63.5000, 82.8074], 5.2559, 0.7008), (1.2699, 3.9685, [58.6210, 67.1250], 2.8347, 1.6479))
	$c_{2}$
$a_{1}$	((0.7629, 5.7215, [48.1073, 69.1250], 2.9191, 1.6970), (1.2983, 4.0572, [59.9310, 68.6250], 2.8980, 1.6847))
$a_{2}$	((0,8.5895, [33.1810, 74.1250], 3.1303, 1.8197), (1.7584, 3.0247, [71.6250, 93.4029], 5.9284, 0.7905))
$a_{3}$	((0.8001, 6.0008, [50.4561, 81.6850], 4.2863, 1.3716), (1.7062, 2.9350, [78.3049, 90.6318], 5.7525, 0.7670))
$a_{4}$	((1.7246, 2.9666, [70.2500, 79.1499], 4.1533, 1.3291), (1.6694, 2.8716, [68, 88.6757], 5.6284, 0.7505))
	$c_{3}$
$a_{1}$	((1.4047, 4.3898, [64.8433, 83.6567], 4.3898, 1.4047), (1.6540, 2.8452, [67.3750, 87.8606], 5.5766, 0.7436))
$a_{2}$	((1.3314, 4.1607, [61.4592, 79.2908], 4.1607, 1.3314), (1.4024, 4.3824, [64.7342, 74.1250], 3.1303, 1.8197))
$a_{3}$	((0.8153, 6.1147, [51.4130, 83.2342], 4.3676, 1.3976), (0.8305, 6.2285, [52.3699, 65.7166], 3.1778, 1.8474))
$a_{4}$	((0.7104, 5.3283, [44.8015, 56.2194], 2.7185, 1.5804), (1.3740, 4.2937, [63.4242, 81.8258], 4.2937, 1.3740))
	$c_{4}$
$a_{1}$	((1.2274, 3.8355, [56.6560, 73.0940], 3.8355, 1.2274), (0,6.7065, [25.9069, 40.2779], 3.4217, 1.0949))
$a_{2}$	((0, 7.7639, [29.9916, 75.4882], 3.9611, 1.2676), (1.6786, 2.8875, [77.0374, 89.1647], 5.6594, 0.7546))
$a_{3}$	((1.3385, 4.1829, [61.7867, 92.2618], 5.8560, 0.7808), (1.5558, 2.6763, [71.4039, 82.6444], 5.2456, 0.6994))
$a_{4}$	((0.8305, 6.2285, [52.3699, 84.7834], 4.4489, 1.4236), (1.6049, 2.7608, [73.6573, 85.2525], 5.4111, 0.7215))

**Table 5:** Z-IVLNS-TTCs evaluation information derived from $d_{2}$.

	$c_{1}$
$a_{1}$	((0, 8.1115, [31.3345, 78.8682], 4.1385, 1.3243), (0.7132, 5.3490, [44.9755, 72.8123], 3.8207, 1.2226))
$a_{2}$	((0.8153, 6.1147, [51.4130, 83.2342], 4.3676, 1.3976), (1.1895, 3.7173, [54.9094, 70.8406], 3.7173, 1.1895))
$a_{3}$	((1.2983, 4.0572, [59.9310, 89.4907], 5.6801, 0.7573), (1.6325, 2.8083, [66.5000, 86.7196], 5.5042, 0.7339))
$a_{4}$	((1.5988, 2.7502, [65.1250, 73.3756], 3.8503, 1.2321), (0,6.7065, [25.9069, 40.2779], 3.4217, 1.0949))
	$c_{2}$
$a_{1}$	((1.2274, 3.8355, [56.6560, 64.8750], 2.7397, 1.5927), (0, 7.9666, [30.7749, 68.7500], 2.9033, 1.6878))
$a_{2}$	((0, 7.8652, [30.3832, 67.8750], 2.8663, 1.6663), (1.4686, 4.5893, [67.7908, 77.6250], 3.2781, 1.9057))
$a_{3}$	((0, 8.2998, [32.0619, 80.6991], 4.2346, 1.3551), (1.3905, 4.3454, [64.1883, 82.8117], 4.3454, 1.3905))
$a_{4}$	((0.7656, 5.7422, [48.2812, 69.3750], 2.9297, 1.7031), (1.3125, 4.1016, [60.5859, 90.4688], 5.7422, 0.7656))
	$c_{3}$
$a_{1}$	((1.2841, 4.0129, [59.2760, 88.5127], 5.6180, 0.7491), (0.8167, 6.1250, [51.5000, 83.3750], 4.3750, 1.4000))
$a_{2}$	((1.1351, 3.5473, [52.3986, 93.1419], 6.9527, 0), (0, 7.8797, [30.4392, 76.6149], 4.0203, 1.2865))
$a_{3}$	((0.8043, 6.0319, [50.7171, 72.8750], 3.0775, 1.7890), (0.8291, 6.2181, [52.2829, 84.6425], 4.4415, 1.4213))
$a_{4}$	((0.8070, 6.0526, [50.8910, 63.8609], 3.0880, 1.7952), (0.7505, 5.6284, [47.3243, 76.6149], 4.0203, 1.2865))
	$c_{4}$
$a_{1}$	((0.6318, 4.7386, [39.8429, 74.6571], 4.7386, 0.6318), (0.6180, 4.6351, [38.9730, 56.0000], 2.3649, 1.3748))
$a_{2}$	((0.7601, 5.7008, [47.9333, 68.8750], 2.9086, 1.6909), (0.6235, 4.6765, [39.3209, 56.5000], 2.3860, 1.3870))
$a_{3}$	((0.7849, 5.8870, [49.4992, 92.7508], 5.8870, 0.7849), (1.3645, 4.2641, [62.9875, 72.1250], 3.0458, 1.7706))
$a_{4}$	((1.2818, 4.0055, [59.1668, 88.3497], 5.6077, 0.7477), (0, 6.6630, [25.7390, 64.7846], 3.3995, 1.0878))

**Table 6:** Z-IVLNS-TTCs evaluation information derived from $d_{3}$.

	$c_{1}$
$a_{1}$	((0.7863, 5.8974, [49.5861, 80.2766], 4.2124, 1.3480), (0.7298, 5.4732, [46.0194, 86.2306], 5.4732, 0.7298))
$a_{2}$	((1.4520, 4.5376, [67.0266, 86.4734], 4.5376, 1.4520), (0.7063, 5.2973, [44.5405, 72.1081], 3.7838, 1.2108))
$a_{3}$	((1.2983, 4.0572, [59.9310, 77.3190], 4.0572, 1.2983), (1.6325, 2.8083, [66.5000, 86.7196], 5.5042, 0.7339))
$a_{4}$	((1.5589, 2.6816, [63.5000, 82.8074], 5.2559, 0.7008), (1.2699, 3.9685, [58.6210, 67.1250], 2.8347, 1.6479))
	$c_{2}$
$a_{1}$	((0, 8.3288, [32.1738, 71.8750], 3.0353, 1.7645), (0,8.2708, [31.9500, 62.3326], 3.0141, 1.7522))
$a_{2}$	((0, 7.9087, [30.5511, 68.2500], 2.8822, 1.6755), (0, 8.4591, [32.6774, 73], 3.0828, 1.7921))
$a_{3}$	((0, 8.2998, [32.0619, 80.6991], 4.2346, 1.3551), (1.3905, 4.3454, [64.1883, 73.5000], 3.1039, 1.8044))
$a_{4}$	((1.7246, 2.9666, [70.2500, 91.6098], 5.8146, 0.7753), (1.6694, 2.8716, [68, 88.6757], 5.6284, 0.7505))
	$c_{3}$
$a_{1}$	((0.7946, 5.9595, [50.1081, 93.8919], 5.9595, 0.7946), (0, 8.2129, [31.7261, 79.8541], 4.1902, 1.3409))
$a_{2}$	((0.7518, 5.6387, [47.4113, 76.7557], 4.0277, 1.2889), (0.6277, 4.7076, [39.5819, 64.0804], 3.3625, 1.0760))
$a_{3}$	((0.8043, 6.0319, [50.7171, 72.8750], 3.0775, 1.7890), (0.8291, 6.2181, [52.2829, 84.6425], 4.4415, 1.4213))
$a_{4}$	((0.9946, 7.4597, [28.8165, 44.8015], 3.8060, 1.2179), (1.3740, 4.2937, [63.4242, 81.8258], 4.2937, 1.3740))
	$c_{4}$
$a_{1}$	((1.1635, 3.6360, [53.7086, 61.5000], 2.5971, 1.5098), (0.6249, 4.6869, [39.4079, 63.7988], 3.3478, 1.0713))
$a_{2}$	((0.7656, 5.7422, [48.2812, 90.4688], 5.7422, 0.7656), (0, 6.3588, [24.5640, 54.8750], 2.3174, 1.3472))
$a_{3}$	((0.7849, 5.8870, [49.4992, 80.1358], 4.2050, 1.3456), (1.3645, 4.2641, [62.9875, 72.1250], 3.0458, 1.7706))
$a_{4}$	((0.8305, 6.2285, [52.3699, 75.2500], 3.1778, 1.8474), (1.6049, 2.7608, [73.6573, 85.2525], 5.4111, 0.7215))

**Table 7:** Collective evaluation information when λ=2.

		Z-IVLNS-TTCs
	$a_{1}$	((1.2168, 4.4066, [56.7071, 74.7552], 3.8348, 1.3940), (1.4297, 3.9600, [49.2397, 63.8645], 4.6853, 1.4714))
$d_{1}$	$a_{2}$	((1.3026, 4.8369, [49.6191, 78.1303], 4.2744, 1.3101), (1.5430, 3.8544, [68.3594, 83.7795], 4.8032, 1.3115))
	$a_{3}$	((1.1775, 4.7521, [55.7408, 87.1546], 5.5172, 1.0210), (1.5480, 3.4834, [67.9018, 82.2284], 5.2321, 0.9813))
	$a_{4}$	((1.4156, 4.1298, [58.6191, 76.6013], 4.4469, 1.2505), (1.5265, 3.3340, [66.2411, 80.8950], 4.9229, 1.0883))
	$a_{1}$	((1.0984, 4.7192, [48.0085, 77.1875], 4.6131, 1.0967), (0.7099, 6.0000, [43.3684, 72.7607], 3.7763, 1.4298))
$d_{2}$	$a_{2}$	((0.8782, 5.5446, [46.5243, 79.1058], 5.0301, 1.2620), (1.1361, 4.9037, [47.1973, 71.1463], 3.6170, 1.4045))
	$a_{3}$	((0.9717, 5.6767, [48.9797, 84.3806], 5.0517, 1.1669), (1.4064, 4.2224, [62.2025, 81.4292], 4.5583, 1.3238))
	$a_{4}$	((1.2501, 4.5117, [56.3359, 74.3489], 4.2847, 1.3376), (0.9869, 5.2816, [39.7434, 67.7439], 4.5323, 1.0384))
	$a_{1}$	((0.8875, 5.6601, [47.1502, 77.7865], 4.5418, 1.3180), (0.5562, 6.4520, [37.8892, 75.7814], 4.4795, 1.1804))
$d_{3}$	$a_{2}$	((1.1146, 5.4384, [50.3974, 80.9841], 4.6011, 1.2942), (0.6001, 5.7185, [37.2731, 66.1940], 3.3471, 1.3279))
	$a_{3}$	((0.9772, 5.6606, [49.0970, 77.7551], 3.9468, 1.4504), (1.3933, 4.2987, [61.8390, 80.0883], 4.4165, 1.3958))
	$a_{4}$	((1.4660, 4.2470, [55.7819, 75.4462], 4.9199, 1.1604), (1.5351, 3.2772, [66.1570, 80.7896], 4.9606, 1.0713))

**Table 8:** $D_{p}$ and $D_{n}$ when λ=2,p=5.

	$a_{1}$ ($D_{p}$, $D_{n}$)	$a_{2}$ ($D_{p}$, $D_{n}$)	$a_{3}$ ($D_{p}$, $D_{n}$)	$a_{4}$ ($D_{p}$, $D_{n}$)
$d_{1}$	0.3324, 0.5224	0.2801, 0.6136	0.2581, 0.6440	0.2545, 0.6035
$d_{2}$	0.3838, 0.5517	0.3751, 0.5541	0.3063, 0.6216	0.3672, 0.5279
$d_{3}$	0.4079, 0.5632	0.3988, 0.5404	0.3077, 0.5927	0.2658, 0.5965

**Table 9:** Ranking when λ=2,p=5.

	$a_{1}$	$a_{2}$	$a_{3}$	$a_{4}$	Ranking
$d_{1}$	0.4274	0.4469	0.4511	0.4290	
$d_{2}$	0.4677	0.4646	0.4640	0.4476	
$d_{3}$	0.4856	0.4696	0.4502	0.4312	
Average distances	0.4602	0.4604	0.4551	0.4359	$a_{2}≻a_{1}≻a_{3}≻a_{4}$

## Data Availability

The data presented in this study are openly available.
